# Kinetoplastid PPEF phosphatases: Dual acylated proteins expressed in the endomembrane system of *Leishmania*

**DOI:** 10.1016/j.molbiopara.2006.11.008

**Published:** 2007-03

**Authors:** Elena Mills, Helen P. Price, Andrea Johner, Jenny E. Emerson, Deborah F. Smith

**Affiliations:** a Wellcome Trust Laboratories for Molecular Parasitology, Centre for Molecular Microbiology and Infection, Imperial College London, London SW7 2AZ, UK; b Immunology and Infection Unit, Department of Biology, University of York, Heslington, York YO10 5YW, UK

**Keywords:** PPEF, Protein Phosphatase with EF-Hands, NMT, *N*-myristoyl transferase, BSF, bloodstream form, PCF, procyclic form, *N*-Myristoylation, Palmitoylation, Protein phosphatases, Bioinformatics

## Abstract

Bioinformatic analyses have been used to identify potential downstream targets of the essential enzyme *N*-myristoyl transferase in the TriTryp species, *Leishmania major*, *Trypanosoma brucei* and *Trypanosoma cruzi*. These database searches predict ∼60 putative *N*-myristoylated proteins with high confidence, including both previously characterised and novel molecules. One of the latter is an *N*-myristoylated protein phosphatase which has high sequence similarity to the Protein Phosphatase with EF-Hand (PPEF) proteins identified in sensory cells of higher eukaryotes. In *L. major* and *T. brucei*, the PPEF-like phosphatases are encoded by single-copy genes and are constitutively expressed in all parasite life cycle stages. The N-terminus of LmPPEF is a substrate for *N*-myristoyl transferase and is also palmitoylated *in vivo*. The wild type protein has been localised to the endocytic system by immunofluorescence. The catalytic and fused C-terminal domains of the kinetoplastid and other eukaryotic PPEFs share high sequence similarity, but unlike their higher eukaryotic relatives, the C-terminal parasite EF-hand domains are degenerate and do not bind calcium.

## 1 Introduction

Protein phosphorylation and dephosphorylation are critical processes in a variety of cellular mechanisms for the detection, transmission, and integration of intra- and extra-cellular signals. In eukaryotes, the extensively studied PPP family of serine/threonine protein phosphatases function in cellular processes as diverse as regulation of the cell-cycle, RNA splicing and T cell activation [1]. PPPs have been divided into three subfamilies, commonly referred to as the ‘Classical’ PPP phosphatases; PP1, PP2A and PP2B [1]. Novel phosphatases that cannot be categorised into these subfamilies include the retinal degeneration C protein (RdgC) from *Drosophila melanogaster* [2]. RdgC homologues are found in a number of eukaryotic species, including *Homo sapiens*, *Mus musculus*, *Gallus gallus* and *Caenorhabditis elegans* [3], [4] but have not been identified in fungi, yeast or plants to date. These novel phosphatases, distinguished by several putative EF-hand motifs within a fused C-terminal domain, have subsequently been termed PPEFs or Protein Phosphatases with EF-Hands [3].

Interestingly, PPEFs exhibit a much narrower tissue distribution than classical PPPs, being restricted to the central nervous system or primary sensory structures in all metazoans studied to date. Thus, Dm RdgC has been principally localised to photoreceptors and the mushroom bodies of the central brain [2], [5] while *C. elegans* CePPEF is highly enriched in primary sensory neurones [6]. *In situ* hybridisation and immunostaining have also localised mammalian PPEF isoforms to sensory structures such as the inner ear, dorsal root ganglia, embryonic brainstem nuclei, photoreceptors and pinealocytes [3], [4]. These findings suggest that the PPEFs have conserved functions in diverse sensory systems and may have a role in development in mammals. The substrates of the RdgC/PPEF phosphatases, however, remain elusive.

**Fig. 1:**
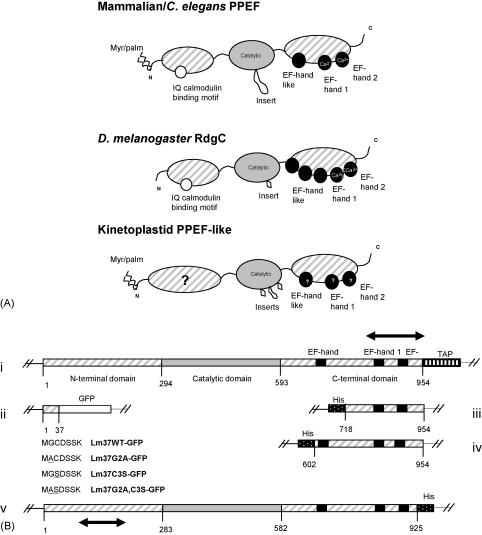
(A) Diagrammatic comparison (not to scale) of the domain organisation of the eukaryotic RdgC/PPEF phosphatases. The three domains for each protein family are indicated: the N-terminal domain that can contain a calmodulin binding motif and/or residues for *N*-myristoylation/palmitoylation (myr/palm); the internal catalytic domain, containing species-specific insertions; the C-terminal domain containing EF-hands. (B) Constructs used in this study: i, LmPPEF-TAP, showing the protein domains as in (A) and the C-terminal TAP tag; the arrow indicates the position of the probe used in blots in Fig. 3A; ii, Lm37WT-GFP and its mutations, Lm37G/A-GFP, Lm37C/S-GFP, Lm37G/A,C/S-GFP; the mutated residues are shown underlined in the first six encoded residues of the protein sequence; iii, LmPPEF-Cterm1, containing an N-terminal His tag and two EF-hands; iv, LmPPEF-Cterm2, containing an N-terminal His tag and three EF-hands; v, TbPPEF-His, containing a C-terminal His tag; the arrow indicates the position of the probe used in blots in Fig. 3B. Amino acids are numbered.

The domain architecture of RdgC/PPEF homologues is highly conserved, consisting of three fused domains (Fig. 1A). The N-terminal regulatory domain often contains myristoyl/palmitoyl acylation motifs and a downstream conserved IQ (isoleucine-glutamine) calmodulin binding domain. The catalytic domain contains several RdgC/PPEF specific sequences/motifs together with insertions of unknown function. The C-terminal domain contains at least two EF-hand motifs that have been shown to bind calcium in HsPPEF-1 and CePPEF [6], [7] and one or more degenerate EF-hand like motifs.

Here, we describe the characterisation of PPEF-like genes in the diverse lower eukaryotes *Leishmania* and *Trypanosoma*, the sole members of this phosphatase family in these parasites. The kinetoplastid PPEFs were identified following a genome-wide search for *N*-myristoylated proteins, carried out to identify downstream targets of the essential gene *N*-myristoyl transferase (NMT) in *Leishmania major*, *Trypanosoma brucei* and *Trypanosoma cruzi*. We show that LmPPEF and TbPPEF are substrates for NMT *in vivo* and that LmPPEF (and probably TbPPEF) can also be palmitoylated *in vivo*. Using immunofluorescence, LmPPEF has been localised to the endocytic system of *Leishmania* parasites, with some accumulation at the flagellar pocket. This location requires downstream regions of the protein in addition to the unique acylated N-terminus. Unlike other members of the RdgC/PPEF family, the EF-hand domains within the C-terminus of LmPPEF are degenerate and do not bind calcium under the experimental conditions used here.

## 2 Materials and methods

### 2.1 PCR amplification and sub cloning

The 2862-bp *LmPPEF* open reading frame (ORF) was amplified from cosmid 1567.3 (gift from Al Ivens) using *Pfu* DNA polymerase (Promega) at 64 °C annealing temperature and the primers LmPPEF_For_ (5′-ATGGGGTGTGACTCATCCAAG-3′) and LmPPEF_Rev_ (5′-TTAGCGACTAGTGCCGAGGC-3′). The amplified *LmPPEF* ORF was cloned into pPCR-Script AMP SK(+) (Stratagene) to generate pLmPPEF.

236-bp and 1056-bp fragments from the 3′ end of the *LmPPEF* ORF (nucleotides 2154–2862 and 1806–2862, respectively) were amplified at 60 °C annealing temperature using primers LmPPEF-Cterm1_For_ (5′-GACGATcatatgCGCATCTGGTAC-3′) and LmPPEF-Cterm1_Rev_ (-5′-TGGCggatccTCTAGCCCTTA-3′) or primers LmPPEF-Cterm2_For_ (5′-ATTAATTTcatatgCAGGTGGTGAGTCTA-3′) and LmPPEF-Cterm2_Rev_ (5′-AATAggatccTTAGCGACTAGTGCC-3′). Cloning sites are shown in lower case. The PCR fragments were digested with *Nde*I/*Bam*HI and cloned into pET-15b (Novagen) generating pLmPPEF-Cterm1 and pLmPPEF-Cterm2, respectively.

The 2775-bp *TbPPEF* ORF was amplified from *T. brucei* genomic DNA at 59 °C annealing temperature, using primers TbPPEF_For_ (5′-CTTACGTTTccatggGTTGCTC-3′) and TbPPEF_Rev_ (5′-CCTCCcTcgagatCTCTCACAAA-3′), digested with *Nco*I/*Xho*I and cloned into pET-33b, generating pTbPPEF. The recombinant TbPPEF protein expressed from this plasmid has an N-terminal myristoylation motif (MGCSTSK).

### 2.2 Parasite culture, membrane fractionation and nucleic acid analysis

*L. major* Friedlin parasites (MHOM/IL/80/Friedlin) were cultured, nucleic acids extracted and DNA/RNA blotting and hybridisation carried out as previously described [8]. For membrane fractionation, mid-log phase parasites (5 × 10^7^) were lysed by sonication on ice in either PBS alone, PBS plus 1 mM CaCl_2_ or PBS plus 1 mM EGTA. Undisrupted cells were removed by two centrifugation steps (500 × *g*, 4 °C, 10 min). Cell lysates were separated into membrane and cytosolic fractions by ultra centrifugation (100,000 × *g*, 4 °C, 1 h). Following separation, membrane fractions were washed twice in PBS and proteins from both fractions analysed by SDS-PAGE and immunoblotting as described [8].

### 2.3 Antibody production and immunoblotting

Expression of N-terminally His-tagged recombinant LmPPEF-Cterm1 was induced by isopropyl-β-d-thiogalactopyranoside (IPTG) in *Escherichia coli* Rosetta (DE3) pLysS (Novagen). Cells were subsequently lysed in 6 M Gu-HCl prior to affinity chromatography using Talon Ni^2+^-nitrilotriacetic acid-agarose (Ni-NTA; BD Biosciences). Eluted protein was precipitated using 10% trichloroacetic acid, air dried and used for immunisation and generation of rabbit polyclonal antiserum (Eurogentech). Partial purification of LmPPEF-specific polyclonal antibodies was carried out using ammonium sulphate precipitation as described [9], followed by affinity purification against purified recombinant LmPPEF-Cterm1 as described [10].

Parasites were lysed in SDS-PAGE gel loading buffer, and immunoblotted as above with purified LmPPEF antiserum (abSK2031, 1:200 dilution), anti-NMT (abSK805, 1:2000 [8]), peroxidase anti-peroxidase (PAP) complex (P-2026, 1:2000, Sigma), or anti-GFP (ab290, 1:10,000, Abcam). Immune complexes were detected using an ECL kit (Amersham Biosciences).

### 2.4 *L. major* episomal expression constructs and parasite transfection

A 111-bp fragment from the 5′ end of the *LmPPEF* ORF (nucleotides1–111) was amplified from pLmPPEF at 58 °C annealing temperature using primers Lm37WT-GFP_For_ (5′-TAAAggatccATGGGGTGTGACTC-3′) and Lm37WT-GFP_Rev_ (5′-TTATAgatatcGCTACAAGTGCGTCG-3′). The fragment was digested with *Bam*HI/*Eco*RV and cloned into pX-GFP [11], generating pLm37WT-GFP. Plasmids pLm37G2A-GFP, pLm37C3S-GFP and pLm37G/A,C/S-GFP were generated as above, using forward primers Lm37G2A-GFP_For_ (5′-TAAAggatccATGGCGTGTGACTC-3′), Lm37C3S-GFP_For_ (5′-TAAAggatccATGGGGTCTGACTC-3′) and Lm37G/A,C/S-GFP_For_ (5′-TAAAggatccATGGCGTCTGACTC-3′), respectively, and the reverse primer Lm37WT-GFP_Rev_.

The 2862-bp *LmPPEF* ORF was amplified from pLmPPEF at 60 °C annealing temperature using primers LmPPEF-TAP_For_ (5′-ATTAATTTcatatgGGGTGTGACTCAT-3′) and LmPPEF-TAP_Rev_ (5′-ATAtctagaCTTGCGGCTAGTGCC-3′), digested with *Nde*I/*Xba*I and cloned into the TAP vector pGL893 (gift from Sebastion Besteiro), generating pLmPPEF-TAP. The recombinant LmPPEF-TAP protein expressed from this plasmid has an *N*-myristoylation motif (MGCDSSK). All constructs used in this study are shown in Fig. 1B.

Mid-log phase *L. major* were electroporated with 20–50 μg of either pLm37WT-GFP, pLm37G2A-GFP, pLm37C3S-GFP, pLm37G/A,C/S-GFP or pLmPPEF-TAP as described [11] and cultures subsequently grown in media supplemented with 1 mg/ml G418 (Life Technologies, Inc.).

### 2.5 Metabolic labelling and immunoprecipitation

Mid-log phase *L. major* promastigotes were metabolically labelled as previously described [11]. Cells were lysed for 1 h at 4 °C in lysis buffer (PBS containing 50 mM Tris, pH 7.5, 150 mM NaCl, 5 mM EDTA, 1% NP-40, 0.5% sodium deoxycholate, 0.1% SDS, 100 μg/ml leupeptin, 500 μg/ml pepstatin, 198 μg/ml 1,10 phenanthroline and 25 μg/ml E64). The lysates were pre-cleared by incubation for 1 h at 4 °C with protein A-coupled Sepharose (Amersham Biosciences). Labelled proteins were then recovered from the supernatant by incubation with either anti-LmPPEF or anti-GFP antibodies overnight at 4 °C. After a second protein A-coupled Sepharose incubation, the beads were collected by centrifugation, washed twice in lysis buffer and proteins removed by boiling in SDS-PAGE gel loading buffer, prior to separation by SDS-PAGE. Detection of radiolabelling was improved using EN^3^HANCE™ Autoradiography Enhancer (Kodak). DTT was omitted from the loading buffer for separation of [9,10-^3^H] palmitate-labelled proteins.

### 2.6 Calcium mobility shift assay

This assay was carried out as described [12]. In brief, proteins were lysed in SDS-PAGE gel loading buffer and separated by SDS-PAGE using either 5 mM CaCl_2_ or 5 mM EGTA in both the stacking and resolving gels. Separated proteins were Coomassie-stained or analysed by immunoblotting.

### 2.7 *N*-Myristoylation co-expression assay

This assay was performed as described [8], [13]. In brief, *E. coli* BL21(DE3) pLysS cells were co-transformed with pTbPPEF and either pNMT [8] or pTbNMT [14]. Expression of recombinant TbPPEF and NMT protein was induced by IPTG in the presence of [^3^H]-myristate (Amersham Biosciences) and, following SDS-PAGE, radiolabelled proteins were detected by autoradiography.

### 2.8 Fluorescence microscopy

For indirect immunofluorescence, *L. major* promastigotes were fixed and processed as previously described [15]. Cells were stained prior to fixation by incubation in 10 μM FM4-64 FX (Invitrogen) in serum-free medium at 26 °C for 10 min. For direct immunofluorescence, parasites were fixed in 4% (w/v) paraformaldehyde at room temperature for 15 min, washed in PBS and immobilised on poly-l-Lysine coated coverslips (4 × 10^6^ cells per cover slip). Slides were mounted using Vectashield plus 4′,6′-diamidino-2-phenylindole (DAPI; Vecta Laboratories).

## 3 Results

### 3.1 The kinetoplastid *N*-myristoylomes

*N*-Myristoylation describes the co-translational addition of the C14:0 fatty acid myristate to the amino-terminal glycine residue of a subset of eukaryotic and viral proteins. Catalysed by the enzyme myristoyl-CoA:protein *N*-myristoyltransferase (NMT), *N*-myristoylation often plays a role in targeting proteins to membrane locations and can additionally be involved in mediating protein–protein interactions and stabilising protein structure. We have previously shown that NMT is essential for viability in kinetoplastid parasites [8] while myristate analogues (non-specific inhibitors of NMT) are lethal to *L. major* promastigotes and bloodstream *T. brucei* [8], [16], [17]. A range of more specific NMT inhibitory compounds has recently been tested and several shown to inhibit *T. brucei* NMT activity *in vitro*, with toxicity to bloodstream *T. brucei* at low μM concentrations [18]. However, the cellular processes contributing to lethality in NMT-depleted cells are as yet unknown, although identification of the essential downstream targets of this enzyme may be crucial to the successful development of NMT as a potential drug target. Few kinetoplastid *N*-myristoylated proteins have been studied in depth to date—the *Leishmania* HASPB and SMP-1 proteins and the *T. brucei* ARL1 protein are exceptions [11], [14], [19].

We used several strategies, all based on the well characterised eukaryotic *N*-myristoylation consensus motif, to search the *L. major*, *T. brucei* and *T. cruzi* genome datasets for putative *N*-myristoylated proteins. The first publicly available prediction algorithm for these proteins, the PDOC00008 PROSITE myristoylation signature [20], is known to generate high numbers of false positive as well as false negative predictions. The more recent *NMT Predictor* program was developed using a set of ‘positive’ amino acid sequences for profile training, substantially reducing the number of false results generated and extending the *N*-myristoylation motif from 6 to 17 amino acids [21], [22]. Further refinement has come with the *Myristoylator* program, based on a neural network model and incorporating an additional ‘negative’ training set of amino acids [23]. We firstly used the ‘relaxed’ version of PROSITE PDOC00008 derived from the *NMT Predictor*, G-{EDRKHPFYW}-x(2)-[STAGCNDEF]-{P} (described in [21]), to probe the kinetoplastid genome datasets (http://www.genedb.org/), thereby reducing the >8000 predicted ORFs to ∼250–300 potentially *N*-myristoylated proteins. The *Myristoylator* prediction algorithm was then applied to this refined data set, generating a final list of predicted *N*-myristoylated proteins within high confidence boundaries (scores: 0.85–1; Table 1; Tables S1–S3, supplementary data). For the three genomes analysed, 62 *N*-myristoylated proteins were predicted in *L. major*, 62 in *T. brucei* and 123 in *T. cruzi*, with the latter higher figure a consequence of the presence of two different haplotypes in the *T. cruzi* CL Brener genome sequence [24]. Predicted *N*-myristoylated proteins contribute 0.5–0.8% of the proteomes of other eukaryotes studied to date [22], [25], and the kinetoplastid data fall well within this range (Table 1). Further analysis of the N-termini of the high confidence kinetoplastid proteins revealed a strong preference (>70% of the total) for serine at position 6 in all three species, with threonine used in a further ∼8% of proteins. In *Saccharomyces cerevisiae*, serine or threonine in these positions allows stabilisation of the peptide–NMT complex through hydrogen bonding with Asp417, Gly418 and His221 in the ScNMT crystal structure [21].

**Table 1:** High confidence kinetoplastid *N*-myristoylated protein predictions

Species	Total no. of predicted proteins encoded by the genome [24], [44], [45]	Total no. of *N*-myristoylated proteins predicted with high confidence	% of proteome predicted to be *N*-myristoylated with high confidence
*Leishmania major*	8,233	62	0.75
*Trypanosoma brucei*	8,164	62	0.76
*Trypanosoma cruzi*	22,570	123	0.54

Of the putative *N*-myristoylated proteins identified with high confidence, 8 were specific to *L. major*, including the dual acylated HASPB [11] and the ADP-ribosylation factor LmARF3 [14], with a similar number of targets specific to *T. brucei* and *T. cruzi* (Tables S1–S3, supplementary data). Other previously characterised *N*-myristoylated proteins included the calpain-like proteins, *T. brucei* CAP 5.5 [26] and *L. major* SMP-1 [19], while a number of putative signal transduction pathway proteins were also identified, including protein kinases and phosphatases (Table 2). However, the largest group of kinetoplastid *N*-myristoylated proteins was that currently characterised as of “unknown function”. A majority of the proteins within this group are conserved between the three Tri-Tryp species.

**Table 2:** Classification of kinetoplastid *N*-myristoylated proteins from high confidence groups

Function	Number in *L. major*	Number in *T. brucei*	Number in *T. cruzi*
Hypothetical proteins (conserved)	33	28	44
Hypothetical proteins (unknown function)	8	14	41
ADP-ribosylation factor (ARFs)	4	3	9
Calpain-like proteins	3	4	9
Ser/Thr protein kinases	2	1	3
Ser/Thr protein phosphatases	2	2	2
Stibogluconate resistance protein	3	0	2
Fatty acyl CoA synthetase	1	0	1
HASPB	1	0	0
Golgi reassembly stacking protein (GRASP)	1	1	2
Other functions	4	9	10

Total	62	62	123

### 3.2 Kinetoplastid PPEF-like protein phosphatases

Analyses of the kinetoplastid genomes have identified a range of kinases and protein phosphatases, some possessing novel motifs and insertions suggesting possible structural and functional differences from their mammalian homologues [24], [27]. Of the phosphatases, only a few of the classical types have been characterised to date: protein phosphatase 2C (PP2C) from *L. chagasi* [28], PP1 from *T. cruzi* [29], PP1, PP2A and PP5 from *T. brucei* [30]. The *N*-myristoylome analysis described above identified a conserved serine/threonine protein PPEF-like phosphatase (hereafter called PPEF) in all three kinetoplastid species: LmjF12.0660 in *L. major*, Tb927.1.4050 in *T. brucei*, Tc00.1047053506529.380/Tc00.1047053510889.80 in *T. cruzi*. These kinetoplastid PPEFs are of similar size and composition: 954 aa, p*I* 8.0, 109.0 kDa in *L. major*; 925 aa, p*I* 7.7, 105.7 kDa in *T. brucei*; 923 aa, p*I* 7.7, 104.8 kDa in *T. cruzi*.

**Fig. 2:**
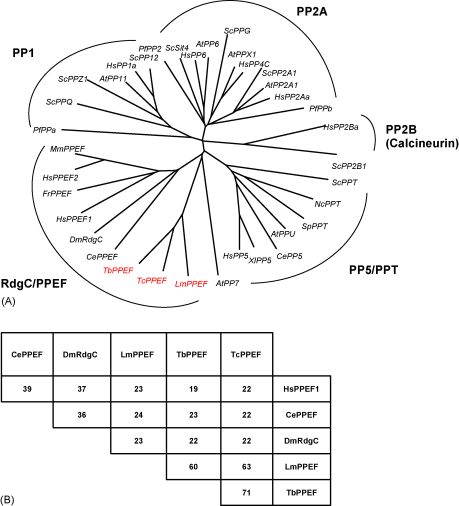
(A) Unrooted dendrogram showing relationships between representative members of the PP1, PP2A, PP2B (calcineurin) subfamilies and RdgC/PP5 subfamilies. CLUSTAL W was used for multiple sequence alignment [31] and the phylogenetic tree generated using the Tree View software version 1.5.2 [32]. Kinetoplastid phosphatases other than those within the RdgC/PPEF family are not included in this analysis. SwissProt or TrEMBL accession numbers: *Plasmodium falciparum* (Pf) PPa, O96914[46]; *Saccharomyces cerevisiae* (Sc) PPQ, P32945[47]; ScPPZ1, P26570[48]. *Arabidopsis thaliana* (At) PP11, P30366[49]. *Homo sapiens* (Hs) PP1a, P62136[50]. ScPP12, P32598[51]. PfPP2, O97259. ScSit4, P20604[52]. HsPP6, O00743[53]. AtPP6, Q9SX52. ScPPG, P32838[54]. AtPPX1, P48529[55]. HsPP4C, P60510[56]. ScPP2A1, P23594[57]. HsPP2a, P67775[58]. PfPPb, O15920[59]. HsPP2Ba, Q08209[60]. ScPP2B1, P23287[61]. ScPPT, P53043[62]. *Neurospora crassa* (Nc) PPT, O14428[63]. *Schizosaccharomyces pombe* (Sp) PPT, O43049. AtPPU, O22662. *Xenopus laevis* (Xl) PP5, O42205[64]. HsPP5, P52041[62]. AtPP7, O49346[65]. CePPEF, O01921[3]. *Drosophila melanogaster* (Dm) RdgC, P40421[2]. HsPPEF 1 + 2, O15253 + O14830[3]. *Fugu rubripes* (Fr) PPEF, Q9W6R4[66]. *Mus musculus* (Mm) PPEF2, O35385[3]. GeneDB accession numbers: LmPPEF, LmjF12.0660. TbPPEF, Tb927.1.4050. TcPPEF1 + 2, Tc00.1047053506529.380, Tc00.1047053510889.80. GenBank accession numbers: CePP5, NP_741697. (B) Amino acid identity between the eukaryotic RdgC/PPEF phosphatases. CePPEF (*Ce*, AAC71139, 707 aa), DmRdgC (*Dm*, AAB00734, 661 aa), HsPPEF-1 (*Hs*, CAA66461, 653 aa), LmPPEF (*Lm*, LmjF12.0660, 954 aa), TbPPEF (Tb, Tb927.1.4050, 925 aa), TcPPEF copies 1 and 2 (Tc, Tc00.1047053506529.380, Tc00.1047053510889.80, 923 aa each).

The phylogenetic relationship between these three proteins, the RdgC/PP5 family and the classical phosphatase groups was investigated by multiple sequence alignment (using CLUSTAL W [31]) and generation of an unrooted tree (using the Tree View software, version 1.5.2 [32]). This analysis confirmed that the trypanosomatid PPEFs are more closely related to the RdgC/PPEF subfamily than to the classical PPP subfamilies, PP1, PP2A or PP2B, and are also separate from the PP5/PPT and PP7 subfamilies (Fig. 2A). Alignments revealed sequence similarity between the kinetoplastid PPEFs and other members of the RdgC/PPEF subfamily (Fig. S1, supplementary data), with the overall percentage of amino acid identity between representative proteins of this group shown in Fig. 2B. While the kinetoplastid proteins share 60–70% identity with each other, they are only ∼20% conserved when compared to their higher eukaryotic relatives. The similarity between these proteins is more apparent, however, when comparing their domain organisation, both at the primary sequence level (Fig. 1A; Fig. S2, supplementary data) and by homology modelling of the catalytic domain of LmPPEF with that of mammalian PP1 for which a high resolution structure is available [33] (Fig. S1, supplementary data). The central catalytic domains of the kinetoplastid and mammalian PPEFs, together with *D. melanogaster* RdgC, share significant sequence similarity (42%), including several RdgC/PP5 specific catalytic motifs [1]. Kinetoplastid PPEFs also share RdgC/PPEF specific mutations found in the conserved SAPNYC motif (common to all PPP phosphatases) that is found within the β12/β13 loop of the catalytic domain (Fig. S2, supplementary data). The first of these, a Pro to Ser substitution at position 3, is found in all members of the Rdg/PP5 family including the kinetoplastid proteins and at least two other protozoan phosphatases, PP1 from *T. cruzi* [29] and PfPPJ, a novel protein phosphatase from *Plasmodium falciparum* [34]. The second substitution within the SAPNYC motif, replacement of the Cys-6 residue by either Tyr, Asp or Asn, is restricted to the RdgC/PPEF sub-family. Overall, the level of conservation within the catalytic domain of LmPPEF, including those residues acting as metal ligands, suggest that this protein and its kinetoplastid orthologues are functional phosphatases (Fig. S1, supplementary data).

At their C-termini, the kinetoplastid PPEFs are more closely related to the C-termini of the RdgC/PPEFs than to any other calcium binding proteins or EF-hand domain-containing enzymes [35]. This observation supports the hypothesis that the catalytic domain of the ancestral form of RdgcC/PPEF fused with an EF-hand Ca^2+^-binding protein prior to the acquisition of the N-terminal domain [1]. At their N-termini, there is little similarity between the kinetoplastid PPEFs and the RdgC/PPEFs except for the presence of putative *N*-myristoylation and palmitoylation motifs, which are found in some but not all members of the RdgC/PPEF phosphatase family.

### 3.3 Genomic organisation and expression of the *L. major* and *T. brucei* PPEFs

The Artemis annotation tool [36] was used to examine the level of gene synteny in the regions flanking the PPEF genes in the *L. major* and *T. brucei* genomes. LmPPEF is found on chromosome 12 in *L. major* while TbPPEF is found on chromosome 1 in *T. brucei* (http://www.genedb.org/). The chromosomal regions surrounding these genes have been analysed previously as part of a wider study, revealing high conservation of gene order [37]. Thus the kinetoplastid PPEFs, including TcPPEF, can be classified as true orthologues.

**Fig. 3:**
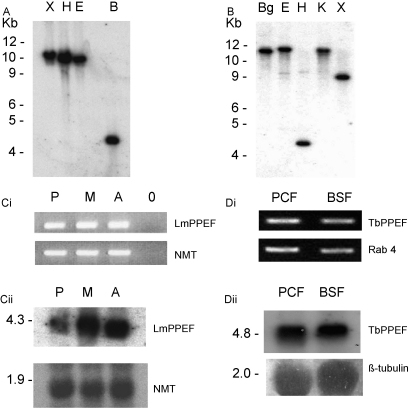
The single copy LmPPEF and TbPPEF genes express RNA constitutively through the parasite life cycles. (A) *L. major* genomic DNA blotting and hybridisation with a LmPPEF probe detects single bands following digestion with *Xho*I (X), *Hin*dIII (H), *Eco*RI (E), *Bam*HI (B). Kb marker sizes are shown. (B) *Trypanosoma brucei* genomic DNA blotting and hybridisation with a TbPPEF probe detects single bands following digestion with *Bgl*II (Bg), *Eco*RI (E), *Hin*dIII (H), *Kpn*I (K), *Xho*I (X). Ci, expression of LmPPEF detected by RT-PCR. Amplification of constitutively expressed NMT was used to monitor equal amounts of cDNA in the reactions. cDNAs from *L. major* procyclics (P), metacyclics (M), amastigotes (A), no cDNA template (0). Cii, LmPPEF RNA expression profile confirmed by blotting and hybridisation of total *L. major* RNA with the LmPPEF probe used in (A), using NMT hybridisation as a loading control. Di, Expression of TbPPEF detected by RT-PCR. Amplification of constitutively expressed Rab4 was used to monitor equal amounts of cDNA in the reactions. cDNAs from *T. brucei* procyclic (PCF) and bloodstream (BSF) forms. Dii, TbPPEF RNA expression profile confirmed by blotting and hybridisation of total *T. brucei* RNA with the TbPPEF probe used in (A), using β-tubulin hybridisation as a loading control.

To confirm the *in silico* gene analysis, genomic DNA blotting and hybridisation were used to show that both LmPPEF and TbPPEF are present as single copy genes in the *L. major* and *T. brucei* genomes, respectively (Fig. 3A and B). RNA expression from these genes in different parasite life cycle stages was initially demonstrated using RT-PCR (Fig. 3Ci and Di). RNA blotting and hybridisation were then used to confirm constitutive expression of single PPEF transcripts (of 4.3 and 4.8 kb, respectively) in insect and mammalian stages of *L. major* and *T. brucei* (Fig. 3Cii and Dii).

**Fig. 4:**
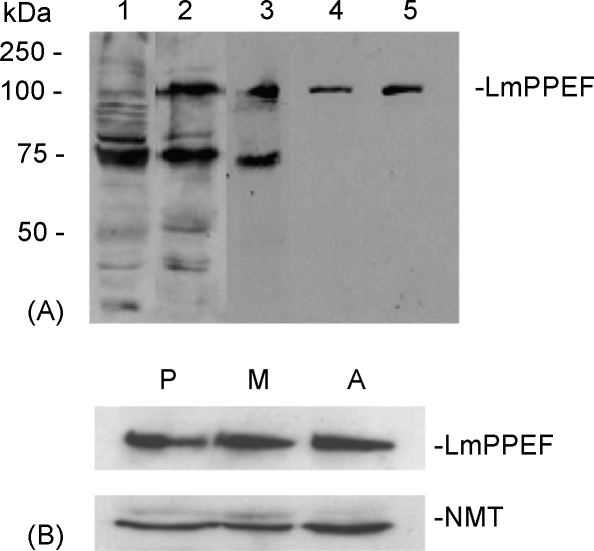
Expression of LmPPEF protein. (A) Affinity purification of LmPPEF antibodies. Using 1 × 10^7^*L. major* procyclics per track, whole cells lysates were immunoblotted with LmPPEF antibodies at each stage of the purification process. Track 1, pre-immunisation test-bleed; track 2, final test-bleed (3 months post-immunisation); track 3, ammonium sulphate purification; track 4, affinity purified LmPPEF antibodies, 1:500 dilution; track 5, affinity purified LmPPEF antibodies, 1:200 dilution. (B) LmPPEF protein expression profile. Using 1 × 10^7^*L. major* procyclics (P), metacyclics (M) or amastigotes (A) per track, whole cell lysates were immunoblotted with the affinity purified anti-LmPPEF from (A). anti-NMT was used on the same blot as a constitutively expressed loading control.

Expression of the LmPPEF protein was analysed by immunoblotting, using affinity-purified antibodies raised against the C-terminal 236 residues of the recombinant protein (expressed from construct LmPPEF-Cterm1, Fig. 1B). These antibodies recognised a single polypeptide band of 109 kDa on immunoblots of whole *L. major* parasite lysates, correlating with the size of the deduced open reading frame of gene LmjF12.0660 (Fig. 4A). Whole cell lysates from *L. major* procyclics, metacyclics and amastigotes were then analysed and a single 109 kDa protein detected in all three life cycle stages (Fig. 4B). However, no signal was detected in *T. brucei* procyclic or bloodstream form parasite lysates using the same anti-LmPPEF (data not shown). This suggests that this C-terminal antibody does not cross react with TbPPEF (despite the 51% amino acid conservation in this domain) and/or that TbPPEF is expressed at low levels during the parasite life cycle.

### 3.4 LmPPEF is not a calcium-binding protein

**Fig. 5:**
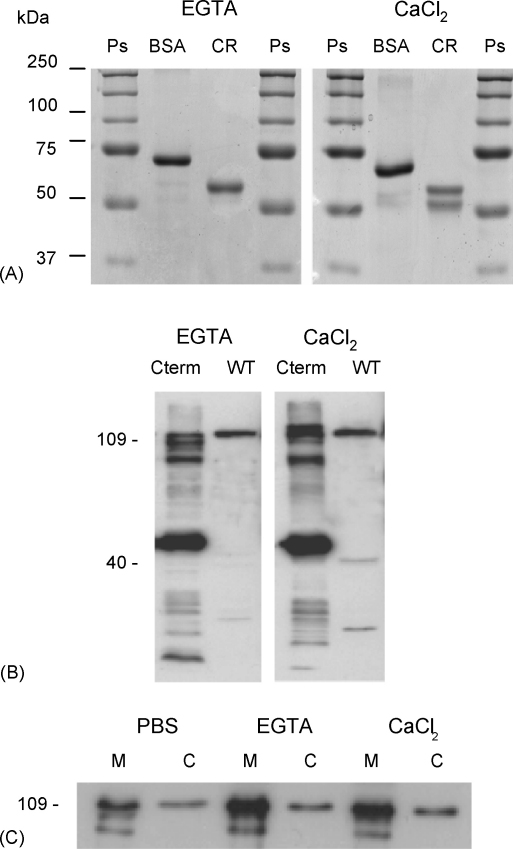
LmPPEF is not a calcium-binding protein. (A) Calcium binding assay: BSA or calreticulin (CR) were separated by SDS-PAGE in the presence or absence of 1 mM EGTA or CaCl_2_ (see Section 2), followed by Coomassie staining. Ps, protein standards. (B) Whole parasite lysates prepared from 1 × 10^7^ LmPPEF-Cterm2 transgenic (Cterm) or wild type (WT) *L. major were* separated as in (A), followed by immunoblotting with anti-LmPPEF. (C) LmPPEF membrane/cytosol distribution following subcellular fractionation in the presence or absence of Ca^2+^. 1 × 10^7^ wild type parasites were lysed in PBS, PBS + 1 mM EGTA or PBS + 1 mM CaCl_2_ before separation into membrane (M) or cytosolic (C) fractions and immunoblotting with anti-LmPPEF.

Given the degeneracy of EF-hand motifs in the C-terminal domains of the kinetoplastid PPEFs, and the absence of N-terminal IQ calmodulin binding motifs, we investigated whether LmPPEF can bind calcium, either as a recombinant or wild type protein, in a mobility shift assay. In this analysis, target and control proteins were separated by electrophoresis through denaturing gels in the presence of either Ca^2+^ or EGTA. Fig. 5A shows the positive (calreticulin) and negative (BSA) control proteins after separation: the migration of BSA was similar in both gel types whereas the migration of calreticulin was significantly altered on electrophoresis through Ca^2+^ as compared to EGTA, with >50% showing a mobility shift. To analyse the behaviour of LmPPEF in this assay, the C-terminal 351 residues, containing all three EF-hand like motifs, were expressed (from construct LmPPEF-Cterm 2) as a 40 kDa N-terminally His-tagged protein in *L. major*. Comparing the mobility of this protein and wild type LmPPEF in whole cell lysates, in the presence of Ca^2+^ or EGTA, revealed no significant differences in migration, suggesting that LmPPEF does not bind calcium (Fig. 5B).

Cell fractionation of total *L. major* proteins was also carried out, to analyse the relative distribution of LmPPEF between membrane and cytoplasm and to investigate whether this distribution was altered in the presence or absence of calcium. Wild type parasites were lysed in either PBS alone, PBS plus 1 mM CaCl_2_ or PBS plus 1 mM EGTA and separated by electrophoresis. Cell fractionation carried out in PBS alone revealed that LmPPEF is predominantly membrane-associated, with ∼20% of the protein detected in the soluble or cytoplasmic fraction (Fig. 5B). The presence or absence of calcium did not alter this membrane versus cytoplasmic distribution, again suggesting that the degenerate EF-hands in LmPPEF are unlikely to bind calcium. A similar conclusion emerged from ^45^Ca overlay experiments with both wild type and recombinant protein (data not shown).

### 3.5 The N-termini of recombinant and wild type kinetoplastid PPEFs are substrates for acylation in vivo

**Fig. 6:**
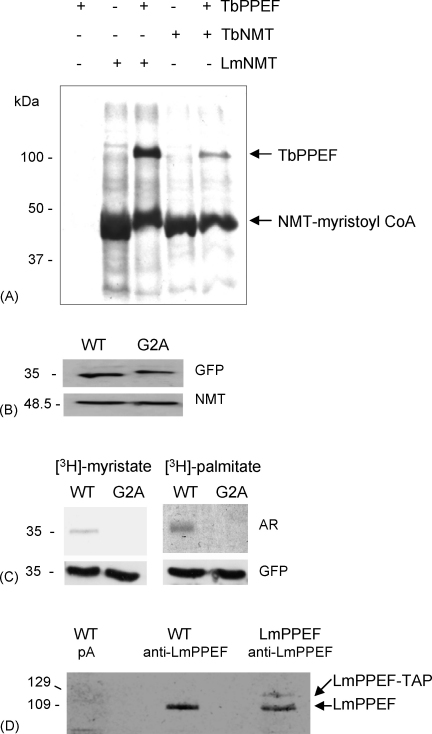
TbPPEF and LmPPEF are acylated *in vivo*. (A) TbPPEF/NMT co-expression assay in *Escherichia coli*. Following induction with IPTG, TbPPEF-His was co-expressed with pET15bTbNMT or pET15b-LmNMT [19] in the presence of [^3^H]-myristate. The radiolabelled products (NMT-myristoyl CoA binary complex and myristoylated TbPPEF) were detected by autoradiography. TbPPEF-His alone (track 1), pET15b-LmNMT alone (track 2), TbPPEF-His/pET15b-LmNMT (track 3), pET15bTbNMT alone (track 4), TbPPEF-His/pET15bTbNMT (track 5). (B) Expression levels of Lm37-GFP fusion proteins in *L. major*. Lysates of 1 × 10^7^*L. major* procyclics overexpressing WT and G2A mutant Lm37-GFP fusion proteins were immunoblotted with anti-GFP. NMT was used as a loading control. (C) *In vivo* radiolabelling of Δ37aaLmPPEF-GFP fusion proteins. *L. major* procyclic overexpressing lines as in (B) were labelled with [^3^H]-myristate or [^3^H]-palmitate (see Section 2), prior to immunoprecipitation with anti-GFP, fractionation, autoradiography (AR) and immunoblotting (GFP). (D) *In vivo* radiolabelling of LmPPEF-TAP fusion protein. *L. major* procyclics (either wild type, tracks 1 and 2, or overexpressing LmPPEF-TAP, track 3) were labelled with [^3^H]-myristate prior to immunoprecipitation with anti-LmPPEF (tracks 2 and 3) or protein A beads alone (pA, track 1), SDS-PAGE and autoradiography.

Given the presence of *N*-myristoylation motifs together with cysteine residues predicted to be palmitoylation sites at the N-termini of kinetoplastid PPEFs (Tables S1–S3, supplementary data), we firstly confirmed that these proteins could act as templates for NMT in an *E. coli* co-expression assay [8]. In these experiments, plasmids expressing NMT and TbPPEF as substrate were co-expressed in the presence of [^3^H]-myristoyl CoA and incorporation of radioactivity into myristoylated product detected by autoradiography following SDS-PAGE (Fig. 6A). Using NMTs from both *L. major* and *T. brucei* with TbPPEF, these results demonstrate radiolabelling of a ∼106 kDa product in the presence of each enzyme, indicative of the transfer of myristate to TbPPEF (as previously demonstrated for HASPA and TbARL1) [8], [14]. While the amount of radiolabelled product appears greater with LmNMT rather than TbNMT in this experiment, this is likely due to an artefact of loading (as indicated by the higher amount of NMT-myristoyl CoA binary complex loaded on to the gel) rather than substrate specificity.

To demonstrate acylation in parasites *in vivo*, metabolic labelling experiments were carried out with *L. major* transgenic lines expressing the first 37 N-terminal residues of PPEF fused with GFP (Lm37WT-GFP, Fig. 1Bii) or a mutated version, in which the Gly in position 2, essential for *N*-myristoylation, is substituted with Ala (Lm37G2A-GFP, Fig. 1Bii). These parasites express equivalent amounts of the two fusion proteins, as indicated by immunoblotting with anti-GFP and anti-NMT (Fig. 6B). Following radiolabelling with [^3^H]-myristate or [^3^H]-palmitate, the fusion proteins were immunoprecipitated using anti-GFP and subjected to SDS-PAGE and autoradiography. This analysis detected a [^3^H]-myristate-labelled protein of 35 kDa, corresponding to the predicted molecular mass of the Lm37WT-GFP protein, which was absent from parasites expressing Lm37G2A-GFP lacking the residue required for *N*-myristoylation (Fig. 6C). Similarly, autoradiography of parasites labelled with [^3^H]-palmitate revealed a single radiolabelled protein of the same molecular mass, corresponding to [^3^H]-palmitate-labelled Lm37WT-GFP. This protein was again absent in parasites expressing Lm37G2A-GFP. These data correlate with similar experiments with the dual acylated HASPB protein, in which loss of the residue required for *N*-myristoylation also prevented palmitoylation, due to mislocalisation of the protein within the cell [11].

The experiments in Fig. 6A and C confirm that TbPPEF and LmPPEF can be acylated *in vivo*, both in a heterologous cell system and in live parasites. To confirm that endogenous LmPPEF is *N*-myristoylated *in vivo*, anti-LmPPEF was used in immunoprecipitations from parasite lysates following metabolic labelling with [^3^H]-myristate. As shown by autoradiography in Fig. 6D, a radiolabelled protein of ∼109 kDa, corresponding to the predicted molecular mass of LmPPEF, was detected in the lysate from wild type *L. major* parasites, while no proteins were detected following precipitation with protein A beads alone. Radiolabelled parasites expressing LmPPEF-TAP (Fig. 1Bi) were also subjected to immunoprecipitation with anti-LmPPEF, generating two detectable [^3^H]-myristate-labelled proteins in the lysate: the smaller corresponding to the 109 kDa native LmPPEF and the larger weaker ∼129 kDa band corresponding to the predicted size of the LmPPEF-TAP fusion protein.

### 3.6 LmPPEF is localised to the endomembrane system of *L. major*

**Fig. 7:**
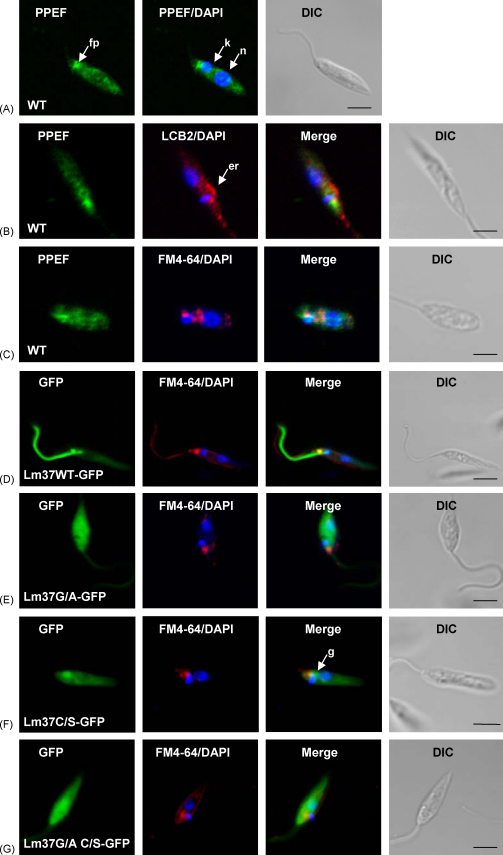
Localisation of LmPPEF. (A–C) wild type (WT) parasites, IFA using anti-LmPPEF (green) or anti-LCB2 (red) and FITC-conjugated anti-rabbit secondary antibody. (D–G) Lm37-GFP expressing parasites. (D) Lm37WT-GFP; (E) Lm37G/A-GFP; (F) Lm37C/S-GFP; (G) Lm37G/A,C/S-GFP. FM4-64 FX staining in red, DAPI staining in blue. Size bars represent 5 μM; n, nucleus; k, kinetoplast; er, endoplasmic reticulum; fp, flagellar pocket; g, Golgi.

Immunofluorescence microscopy with the affinity-purified LmPPEF antibody was used to investigate the location of LmPPEF in procyclic *L. major* (Fig. 7A). Overlay of the fluorescent signal with DAPI (for identification of the nucleus and kinetoplast) indicated a punctate distribution of signal within the cytosol, reminiscent of the endocytic system, together with some concentration in the region of the flagellar pocket. These observations are consistent with the predominantly membrane association of LmPPEF revealed by cellular fractionation (Fig. 5). Counter-staining with anti-LCB2, which recognises an ER-resident sub-unit of serine palmitoyltransferase [38] showed no overlap with PPEF (Fig. 7B) while labelling with the endocytic lipophilic tracer, FM4-64, demonstrated some co-localisation with the flagellar pocket and endosomes (Fig. 7C). PPEF staining was excluded from the nucleus, plasma membrane and flagellum.

We attempted to express and analyse GFP-fusions with either full-length wild type LmPPEF or the N-terminal 473 residues alone (lacking the C-terminal EF-hands). However, these constructs did not express after transfection into *L. major*, probably due to the large sizes of the recombinant proteins generated. As an alternative approach, given that the N-terminus of LmPPEF is a target for acylation (Fig. 6), the N-terminal GFP transgenic parasite lines (see Fig. 1B) were used to investigate the role of these modifications in intracellular localisation of the fusion proteins. Counter-staining with FM4-64 was also utilized to detect the flagellar pocket in these analyses. Transfected parasites expressing the first 37 residues of LmPPEF fused to GFP (Lm37WT-GFP) targeted the fusion protein predominantly to the flagellum and the flagellar pocket, with weak staining only in the cell body (Fig. 7D). Loss of the *N*-myristoylation site by mutation of Gly to Ala (Lm37G2A-GFP) resulted in homogeneous distribution of cytosolic fluorescence throughout the parasite (Fig. 7E), in a pattern almost identical to the localisation of GFP alone (data not shown). Separate mutation of the putative palmitoylation site alone, by substituting Cys-3 with Ser (Lm37C3S-GFP), resulted in concentrated signal in a region adjacent to the flagellar pocket most likely to be the Golgi (Fig. 7F). Expression of the double mutant Lm37G2A, C3S-GFP, lacking both acylation sites, caused the fusion protein to remain localised within the cytosol (Fig. 7G). These observations correlate with those previously observed using similar N-terminal mutations of the *L. major* HASPB protein [11], suggesting that the wild type fusion protein (Lm37WT-GFP) requires *N*-myristoylation in the cytosol to reach the Golgi region, where palmitoylation further modifies the protein for trafficking to other locations, predominantly the flagellum. Clearly, the localisations of these GFP fusion proteins are distinct from the endocytic location of wild type LmPPEF, indicating that other regions and/or signals within this large protein are important for subcellular localisation. More generally, these data suggest that N-terminal dual acylation might act as a primary signal in targeting proteins to the flagellum in *Leishmania*, as exemplified by HASPB, SMP-1 and PPEF [11], [19].

## 4 Discussion

In this paper, as part of a wider study of the downstream targets of NMT, we describe the PPEF-like protein phosphatases that are encoded by single copy genes in three kinetoplastid species. These phosphatases were identified following *in silico* genome analysis that predicted a subset of kinetoplastid proteins as “high confidence” substrates for NMT. Within this subset were several proteins already confirmed as *N*-myristoylated in *L. major* or *T. brucei*, together with a number of species-specific molecules and a larger group of proteins found in all three species, many of no known function. We predict that all of these “high confidence” putative substrates are acylated *in vivo* but do not exclude other “medium confidence” proteins as candidates for NMT modification.

LmPPEF and TbPPEF, studied in detail here, are constitutively expressed, membrane-associated acylated proteins. LmPPEF is found in the endocytic system and the flagellar pocket. Conservation of key features and residues within the catalytic domains, which have been modelled against the mammalian PP1 catalytic region, suggest that these proteins are active enzymes (although this has not yet been demonstrated biochemically). Unlike these conserved central regions, however, divergence in the N- and C-terminal domains has led to loss of the IQ calmodulin binding motif and degeneration of the EF-hands, features that characterise the higher eukaryotic PPEFs [39], [40]. Thus, there is no evidence that the kinetoplastid proteins are regulated by cytoplasmic Ca^2+^ levels and/or calmodulin, in contrast to the demonstrated interactions of both human PPEF and *Drosophila* RdgC with these regulators [39], [40]. The analysis in Fig. 5 would support this conclusion.

Although PPEF-like proteins have been characterised in a number of species, the functions of these unusual phosphatases are not well understood. The *Drosophila* RdgC protein is implicated in dephosphorylation of rhodopsin, a G protein-coupled receptor (GPCR) that initiates vertebrate and invertebrate phototransduction. However, mutant mice with targeted disruptions in each of their two PPEF genes show no retinal degeneration and normal rhodopsin dephosphorylation kinetics, suggesting interspecies functional differences despite high similarities in protein sequence [41].

Completion of the Tri-Tryp genome projects has confirmed earlier predictions that these parasites have ‘stream-lined’ signal transduction mechanisms as compared to their higher eukaryotic multicellular counterparts [42]. The lack of several classes of signalling molecules, including serpentine receptors, heterotrimeric G proteins and most classes of catalytic receptors, contrasts with the presence of a large and diverse family of kinase and phosphatases, suggesting complex cellular interactions [43]. While the conservation and expression of PPEF genes in the Kinetoplastida strongly suggest a functional role for these unusual phosphatases, it is clear that this is unlikely to be similar to that demonstrated for the RdgC protein. We have perturbed *TbPPEF* expression by inducible RNAi in both bloodstream and procyclic stages of *T. brucei*, which results in a partial growth defect under normal culture conditions (data not shown). However, it cannot be discounted in these experiments that a more extreme phenotype is masked by sufficient residual expression to support enzyme activity. Functional analysis of conditional gene deletion mutants will be required, together with substrate identification, to delineate roles for the kinetoplastid PPEFs in parasite viability.

## References

[1] Andreeva A.V., Kutuzov M.A. (2001). PPP family of protein Ser/Thr phosphatases: two distinct branches?. Mol Biol Evol.

[2] Steele F.R., Washburn T., Rieger R. (1992). Drosophila retinal degeneration C (rdgC) encodes a novel serine/threonine protein phosphatase. Cell.

[3] Sherman P.M., Sun H., Macke J.P. (1997). Identification and characterization of a conserved family of protein serine/threonine phosphatases homologous to Drosophila retinal degeneration C. Proc Natl Acad Sci USA.

[4] Montini E., Rugarli E.I., Van de Vosse E. (1997). A novel human serine-threonine phosphatase related to the Drosophila retinal degeneration C (rdgC) gene is selectively expressed in sensory neurons of neural crest origin. Hum Mol Genet.

[5] Kurusu M., Nagao T., Walldorf U. (2000). Genetic control of development of the mushroom bodies, the associative learning centers in the Drosophila brain, by the eyeless, twin of eyeless, and Dachshund genes. Proc Natl Acad Sci USA.

[6] Ramulu P., Nathans J. (2001). Cellular and subcellular localization, N-terminal acylation, and calcium binding of *Caenorhabditis elegans* protein phosphatase with EF-hands. J Biol Chem.

[7] Huang X., Honkanen R.E. (1998). Molecular cloning, expression, and characterization of a novel human serine/threonine protein phosphatase, PP7, that is homologous to Drosophila retinal degeneration C gene product (rdgC). J Biol Chem.

[8] Price H.P., Menon M.R., Panethymitaki C. (2003). Myristoyl-CoA:protein *N*-myristoyltransferase, an essential enzyme and potential drug target in kinetoplastid parasites. J Biol Chem.

[9] Harlow E., Lane D. (1988). Antibodies: a laboratory manual.

[10] Ritter K. (1991). Affinity purification of antibodies from sera using polyvinylidenedifluoride (PVDF) membranes as coupling matrices for antigens presented by autoantibodies to triosephosphate isomerase. J Immunol Methods.

[11] Denny P.W., Gokool S., Russell D.G. (2000). Acylation-dependent protein export in Leishmania. J Biol Chem.

[12] Garrigos M., Deschamps S., Viel A. (1991). Detection of Ca^2+^-binding proteins by electrophoretic migration in the presence of Ca^2+^ combined with ^45^Ca^2+^ overlay of protein blots. Anal Biochem.

[13] Duronio R.J., Towler D.A., Heuckeroth R.O. (1989). Disruption of the yeast *N*-myristoyl transferase gene causes recessive lethality. Science.

[14] Price H.P., Panethymitaki C., Goulding D. (2005). Functional analysis of TbARL1, an *N*-myristoylated Golgi protein essential for viability in bloodstream trypanosomes. J Cell Sci.

[15] Denny P.W., Lewis S., Tempero J.E. (2000). Leishmania RAB7: characterisation of terminal endocytic stages in an intracellular parasite. Mol Biochem Parasitol.

[16] Doering T.L., Raper J., Buxbaum L.U. (1991). An analog of myristic acid with selective toxicity for African trypanosomes. Science.

[17] Doering T.L., Lu T., Werbovertz K.A. (1994). Toxicity of myristic acid analogs toward African trypanosomes. Proc Natl Acad Sci USA.

[18] Panethymitaki C., Bowyer P.W., Price H.P. (2006). Characterisation and selective inhibition of Myristoyl CoA: protein *N*-myristoyl transferase from *Trypanosoma brucei* and Leishmania major. Biochem J.

[19] Tull D., Vince J.E., Callaghan J.M. (2004). SMP-1, a member of a new family of small myristoylated proteins in kinetoplastid parasites, is targeted to the flagellum membrane in Leishmania. Mol Biol Cell.

[20] Falquet L., Pagni M., Bucher P. (2002). The PROSITE database, its status in 2002. Nucleic Acids Res.

[21] Maurer-Stroh S., Eisenhaber B., Eisenhaber F. (2002). N-terminal *N*-myristoylation of proteins: refinement of the sequence motif and its taxon-specific differences. J Mol Biol.

[22] Maurer-Stroh S., Eisenhaber B., Eisenhaber F. (2002). N-terminal *N*-myristoylation of proteins: prediction of substrate proteins from amino acid sequence. J Mol Biol.

[23] Bologna G., Yvon C., Duvaud S. (2004). N-terminal myristoylation predictions by ensembles of neural networks. Proteomics.

[24] El-Sayed N.M., Myler P.J., Bartholomeu D.C. (2005). The genome sequence of *Trypanosoma cruzi*, etiologic agent of Chagas’ disease. Science.

[25] Maurer-Stroh S, Gouda M, Novatchkova M, et al. MYRbase: analysis of genome-wide glycine myristoylation enlarges the functional spectrum of eukaryotic myristoylated proteins. Genome Biol 2004;5:R21,1–16. 10.1186/gb-2004-5-3-r21 PMC395771 15003124

[26] Hertz-Fowler C., Ersfeld K., Gull K. (2001). CAP5.5, a life-cycle-regulated, cytoskeleton-associated protein is a member of a novel family of calpain-related proteins in *Trypanosoma brucei*. Mol Biochem Parasitol.

[27] Doerig C., Meijer L., Mottram J.C. (2002). Protein kinases as drug targets in parasitic protozoa. Trends Parasitol.

[28] Burns J.M., Parsons M., Rosman D.E. (1993). Molecular cloning and characterization of a 42-kDa protein phosphatase of *Leishmania chagasi*. J Biol Chem.

[29] Orr G.A., Werner C., Xu J. (2000). Identification of novel serine/threonine protein phosphatases in *Trypanosoma cruzi*: a potential role in control of cytokinesis and morphology. Infect Immun.

[30] Erondu N.E., Donelson J.E. (1991). Characterization of trypanosome protein phosphatase 1 and 2A catalytic subunits. Mol Biochem Parasitol.

[31] Thompson J.D., Higgins D.G., Gibson T.J. (1994). CLUSTAL W: improving the sensitivity of progressive multiple sequence alignment through sequence weighting, position-specific gap penalties and weight matrix choice. Nucleic Acids Res.

[32] Page R.D. (1996). TreeView: an application to display phylogenetic trees on personal computers. Comput Appl Biosci.

[33] Goldberg J., Huang H.B., Kwon Y.G. (1995). Three-dimensional structure of the catalytic subunit of protein serine/threonine phosphatase-1. Nature.

[34] Dobson S., Bracchi V., Chakrabarti D. (2001). Characterization of a novel serine/threonine protein phosphatase (PfPPJ) from the malaria parasite, *Plasmodium falciparum*. Mol Biochem Parasitol.

[35] Andreeva A.V., Kutuzov M.A. (1999). RdgC/PP5-related phosphatases: novel components in signal transduction. Cell Signal.

[36] Rutherford K., Parkhill J., Crook J. (2000). Artemis: sequence visualization and annotation. Bioinformatics.

[37] Ghedin E., Bringaud F., Peterson J. (2004). Gene synteny and evolution of genome architecture in trypanosomatids. Mol Biochem Parasitol.

[38] Denny P.W., Goulding D., Ferguson M.A., Smith D.F. (2004). Sphingolipid-free Leishmania are defective in membrane trafficking, differentiation and infectivity. Mol Microbiol.

[39] Lee S.J., Montell C. (2001). Regulation of the rhodopsin protein phosphatase, RDGC, through interaction with calmodulin. Neuron.

[40] Kutuzov M.A., Solov’eva O.V., Andreeva A.V. (2002). Protein Ser/Thr phosphatases PPEF interact with calmodulin. Biochem Biophys Res Commun.

[41] Ramulu P., Kennedy M., Xiong W.H. (2001). Normal light response, photoreceptor integrity, and rhodopsin dephosphorylation in mice lacking both protein phosphatases with EF hands (PPEF-1 and PPEF-2). Mol Cell Biol.

[42] Parsons M., Ruben L. (2000). Pathways involved in environmental sensing in trypanosomatids. Parasitol Today.

[43] El-Sayed N.M., Myler P.J., Blandin G. (2005). Comparative genomics of trypanosomatid parasitic protozoa. Science.

[44] Berriman M., Ghedin E., Hertz-Fowler C. (2005). The genome of the African trypanosome *Trypanosoma brucei*. Science.

[45] Ivens A.C., Peacock C.S., Worthey E.A. (2005). The genome of the kinetoplastid parasite, Leishmania major. Science.

[46] Li J.L., Baker D.A. (1998). A putative protein serine/threonine phosphatase from *Plasmodium falciparum* contains a large N-terminal extension and five unique inserts in the catalytic domain. Mol Biochem Parasitol.

[47] Chen M.X., Chen Y.H., Cohen P.T. (1993). PPQ, a novel protein phosphatase containing a Ser + Asn-rich amino-terminal domain, is involved in the regulation of protein synthesis. Eur J Biochem.

[48] Posas F., Casamayor A., Morral N., Arino J. (1992). Molecular cloning and analysis of a yeast protein phosphatase with an unusual amino-terminal region. J Biol Chem.

[49] Nitschke K., Fleig U., Schell J., Palme K. (1992). Complementation of the cs dis2-11 cell cycle mutant of *Schizosaccharomyces pombe* by a protein phosphatase from *Arabidopsis thaliana*. Embo J.

[50] Song Q., Khanna K.K., Lu H., Lavin M.F. (1993). Cloning and characterization of a human protein phosphatase 1-encoding cDNA. Gene.

[51] Ohkura H., Kinoshita N., Miyatani S., Toda T., Yanagida M. (1989). The fission yeast dis2 + gene required for chromosome disjoining encodes one of two putative type 1 protein phosphatases. Cell.

[52] Arndt K.T., Styles C.A., Fink G.R. (1989). A suppressor of a HIS4 transcriptional defect encodes a protein with homology to the catalytic subunit of protein phosphatases. Cell.

[53] Bastians H., Ponstingl H. (1996). The novel human protein serine/threonine phosphatase 6 is a functional homologue of budding yeast Sit4p and fission yeast ppe1, which are involved in cell cycle regulation. J Cell Sci.

[54] Posas F., Clotet J., Muns M.T., Corominas J., Casamayor A., Arino J. (1993). The gene PPG encodes a novel yeast protein phosphatase involved in glycogen accumulation. J Biol Chem.

[55] Perez-Callejon E., Casamayor A., Pujol G., Clua E., Ferrer A., Arino J. (1993). Identification and molecular cloning of two homologues of protein phosphatase X from *Arabidopsis thaliana*. Plant Mol Biol.

[56] Brewis N.D., Cohen P.T. (1992). Protein phosphatase X has been highly conserved during mammalian evolution. Biochim Biophys Acta.

[57] Sneddon A.A., Cohen P.T., Stark M.J. (1990). *Saccharomyces cerevisiae* protein phosphatase 2A performs an essential cellular function and is encoded by two genes. Embo J.

[58] Stone S.R., Mayer R., Wernet W., Maurer F., Hofsteenge J., Hemmings B.A. (1988). The nucleotide sequence of the cDNA encoding the human lung protein phosphatase 2A alpha catalytic subunit. Nucleic Acids Res.

[59] Li J.L., Baker D.A. (1997). Protein phosphatase beta, a putative type-2A protein phosphatase from the human malaria parasite *Plasmodium falciparum*. Eur J Biochem.

[60] Muramatsu T., Kincaid R.L. (1993). Molecular cloning of a full-length cDNA encoding the catalytic subunit of human calmodulin-dependent protein phosphatase (calcineurin A alpha). Biochim Biophys Acta.

[61] Cyert M.S., Kunisawa R., Kaim D., Thorner J. (1991). Yeast has homologs (CNA1 and CNA2 gene products) of mammalian calcineurin, a calmodulin-regulated phosphoprotein phosphatase. Proc Natl Acad Sci USA.

[62] Chen M.X., McPartlin A.E., Brown L., Chen Y.H., Barker H.M., Cohen P.T. (1994). A novel human protein serine/threonine phosphatase, which possesses four tetratricopeptide repeat motifs and localizes to the nucleus. Embo J.

[63] Yatzkan E., Yarden O. (1997). ppt-1, a *Neurospora crassa* PPT/PP5 subfamily serine/threonine protein phosphatase. Biochim Biophys Acta.

[64] Ollendorff V., Donoghue D.J. (1997). The serine/threonine phosphatase PP5 interacts with CDC16 and CDC27, two tetratricopeptide repeat-containing subunits of the anaphase-promoting complex. J Biol Chem.

[65] Andreeva A.V., Evans D.E., Hawes C.R., Bennett N., Kutuzov M.A. (1998). PP7, a plant phosphatase representing a novel evolutionary branch of eukaryotic protein Ser/Thr phosphatases. Biochem Mol Biol Int.

[66] Brunner B., Todt T., Lenzner S. (1999). Genomic structure and comparative analysis of nine Fugu genes: conservation of synteny with human chromosome Xp22.2-p22.1. Genome Res.

