# Supplementary material for: Kinetoplastid PPEF phosphatases: Dual acylated proteins expressed in the endomembrane system of Leishmania
Source: Mol Biochem Parasitol. 2007 Mar;152(1):22–34. doi: 10.1016/j.molbiopara.2006.11.008 (PMC1885993; doi:10.1016/j.molbiopara.2006.11.008)
Supplement: Supplementary file 1 [file mmc1.pdf]

Figure S1. Alignment of the kinetoplastid PPEFs with RdgC/PPEF proteins from other eukaryotes

Key: Hs: *Homo sapiens*. **Mm:** *Mus musculus*. **Dm:** *Drosophila melanogaster*. **Ce:** *Caenorhabditis elegans*. **Tc:** *Trypanosoma cruzi*. **Tb:** *Trypanosoma brucei.* **Lm:** *Leishmania major.*

Residues in red correspond to the phosphatase catalytic region. Regions block highlighted in red correspond to residues matching that of the EF-hand consensus motif. Red asterisks highlight residue positions within the EF-hand motif essential for calcium ion coordination. Residues block highlighted in blue correspond to the calmodulin IQ-type motif (X = any residue). Boxed residues indicate distinctive regions of RdgC/PPEFs absent from other PPP enzymes.

```
HsPPEF2         ------------------------------------------------------------
MmPPEF          ------------------------------------------------------------
DmRdgC          ------------------------------------------------------------
CePPEF          ----------------MGCGPSSGRQNPSTELKKSTRATTTTTSSSQRNNYNDNNQNTSS 44
TcPPEF          MGCTNTKESSERG----GGNASELGK--------GGRVGLT----------GVLAANGLH 38
TbPPEF          MGCSTSKGDPLRTNKGAGDGAGSLRNP------PGGRWSQT----------DYPDVG--- 41
LmPPEF          MGCDSSKE----GVASNTASASSRGSRNCAEEEKGGRRTCSN---DSTEVKGKSGASGAH 53


HsPPEF2         ------MGSGTSTQHHFAFQNAER------------------------------------ 18
MmPPEF          ------MGSSSSTQHHFAFQNAEK------------------------------------ 18
DmRdgC          --------------------MDEN------------------------------------ 4
CePPEF          SSGNKKESSSSSKQHSSKKSKKSNSK------------------------KNRSPSPQPQ 80
TcPPEF          GGGSANDQVHWPTCHFCGKRIPEKEYNAHTVMCDEREVTCWNSWCRQIVKQGMLHNHLEE 98
TbPPEF          ------NQRHLPQCHFCKRFIAEKEYNAHTVMCDEREMTCWNSWCRQMVKQGMLSKHLEE 95
LmPPEF          NGGAMADQSRWPTCHFCHRKVPDKDYNAHTVMCDEREVTCWNSWCRQIVRQGDLQKHLEE 113
                                      ..
                        IQXXXRXXXXR
HsPPEF2         -AFKAAALIQRWYRRYVARLEMRRRCTWS--------------IFQSIEYAGQQDQVKLH 63
MmPPEF          -AFKAAALIQRWYRRYMARLEMRRRCTWN--------------IFQSIEYAGQQDQVKLH 63
DmRdgC          -AIRAAIFIQKWYRRHQARREMQRRCNWQ--------------IFQNLEYASEQDQAELY 49
CePPEF          LTIKSAILIQKWYRRCEARLEARRRATWQ--------------IFTALEYAGEQDQLKLY 126
TcPPEF          CAKKQRALCYKCGADLLATELQAHRDGCQPQKCTSCGELCITRIMKWCPDNFITRVPIMH 158
TbPPEF          CAKNQRALCYKCGTEVLASDLQVHRDTCQPKKCSACGELCITRILSWCPHNFG-RIGATQ 154
LmPPEF          CGRRQKAICPKCGAEILAIEMLAHRDSCQPKPCPQCGELCITRIHKWCPKTILAKVKFIN 173
                   .   :  :      *     :*   .              *

HsPPEF2         DFFSYLMDHFIP------------------SSHND------------------------- 80
MmPPEF          EFFSYLVDHFTP------------------SSHHE------------------------- 80
DmRdgC          KFFNDLIKHMPQAAGRKNQYQGSAH----VSVLDD------------------------- 80
CePPEF          DFFADVIRAMAEENGKGGVENGRNSPLMSALSHYA------------------------- 161
TcPPEF          GPFATEVLLARYVKGGAQNKSGGAAPVALVS-----YNVARMQLLWRWTKAKSYLEEAIF 213
TbPPEF          GPFATEALSKKYLSGGGRPK--GASSVHNNSPPHVNFNVARMQLLWRWIKTKSIIEETLF 212
LmPPEF          GPFATEALQRRFMPPG---KEKDPRAKLEYS-------VARMQLLWRWIKCKGMIEETIF 223
                  *
HsPPEF2         -----RDFLTRIFTEDRFAQDSEMKKCSDYES------------IEVPDSYTGPSLSFPL 123
MmPPEF          -----RDFLNRMFTEERFAQDVETEEGGDFES------------IEVPDSYTGPRLSFPL 123
DmRdgC          -----KDDLVEEFGDIVNAK------------------------IELPIRKN-------- 103
CePPEF          -----KPSLMDSEGETVKKMLEDTSPTN----------------VDIDRNYKGPTLSLPL 200
TcPPEF          RLTSKEMNLKKEGFAIFKALDKVSDSHVLEPRRSRSILQTPVVAPAISSHYFPTRMAEPI 273
TbPPEF          RVMSREMDLKKEGFAIFKALDKVNESHVLAPKRSRSILQSPVVAPAVSSHYFPTRSSDPI 272
LmPPEF          RSVYKEMDLKKEGFAIFKAHDKVNEQHVMAPKRSRSVIQAPVVAPATSSHYFPVNTNIPI 283
                     .  *

HsPPEF2         LPDHATALVEAFRLK--QQLHARYVLNLLYETKKHLVQLPNINRVSTCYSEEIT------ 175
MmPPEF          LPDHATALVEAFRLR--QQLHARYVLNLLYETRKHLAQLPNINRVSTCYSEEVT------ 175
DmRdgC          ---HIDLLIDVFRKKRGNRLHPKYVALILREAAKSLKQLPNISPVSTAVSQQVT------ 154
CePPEF          DKPQVAKMIEAFKVN--KVLHPKYVLMILHEARKIFKAMPSVSRISTSISNQVT------ 252
TcPPEF          TIEVVQRLINDLTDH--VLLPYPAAWRVFTDAMSHLNTMPNVVRLSPPVGARVSNGRINQ 331
TbPPEF          TLDVVSRLMKDLSEH--ILPPYPAVWRVFTDAMCYLNTMPNVVLLSPPLGARVVNGRVNQ 330
LmPPEF          TLHHVEQMIKDIKNR--VLLPYPAAWRVFTDAMNHLNTMPNVVRVSPAVGARVSNGRVIQ 341
                       ::. :  .         .  :: ::   :  :*.:  :*.  . .:
```

```
HsPPEF2         -----VCGDLHGQLDDLIFIFYKNGLPSPERSYVFNGDFVDRGKDSVEILMILFAFMLVY 230
MmPPEF          -----VCGDLHGQLDDLIFIFYKNGLPSPERAYVFNGDFVDRGKDSVEVLMVLFAFMLVY 230
DmRdgC          -----VCGDLHGKLDDLLVVLHKNGLPSSSNPYVFNGDFVDRGKRGLEVLLLLLSLYLAF 209
CePPEF          -----ICGDLHGKFDDLCIILYKNGYPSVDNPYIFNGDFVDRGGQSIEVLCVLFALVIVD 307
TcPPEF          GSKVVVVGDLHGQLADLLHILKECGMPNEGTYYIFNGDFVDRGANGVEVLLILFSLMLAC 391
TbPPEF          GSKVVVVGDLHGQLADLLHILKECGMPNDSTYYIFNGDFVDRGPNGVEVLLIIFSLMLAC 390
LmPPEF          GCKVVVVGDLHGQIADLLHILKESGMPSESNYYVFNGDYVDRGACGVEIILILFSLMLAC 401
                     : *****:: **  :: : * *.    *:****:****  .:*:: ::::: :.

HsPPEF2         PKEFHLNRGNHEDHMVNLRYGFTKEVMNKYKVHGKEILRTLQDVFCWLPLATLIDEKVLI 290
MmPPEF          PKEFHLNRGNHEDHLVNLRYGFTKEVMHKYKIHGKKILRTLQDVFCWLPLATLVDEKVLV 290
DmRdgC          PNAVFLNRGNHEDSVMNARYGFIREVESKYPRNHKRILAFIDEVYRWLPLGSVLNSRVLI 269
CePPEF          PMSIYLNRGNHEDHIMNLRYGFIKELSTKYKDLSTPITRLLEDVFSWLPIATIIDRDIFV 367
TcPPEF          PKYVTLNRGNHECDYMNDEYGFDVEVSTKYDRN---VFRLVQRCFCALPLATIIGKKVFV 448
TbPPEF          PKFVTLNRGNHECDYMNEEYGFDVEVSTKYDRN---VFRLIQRCFCAMPLATIIGRKVFV 447
LmPPEF          PKYVTLNRGNHECDYMNDEYGFDVEVSTKYDRN---VFRLIQRCFCALPLATLIGGKIFV 458
                *   . *******    :* .***  *:   **       :    ::  :  :*:.:::.  :::

HsPPEF2         LHGGVSDITDLELLDKIERSKIVSTMRCKTRQKSEKQMEEKRRANQKSSAQGPIPWFLPE 350
MmPPEF          LHGGVSDKTDLELLAKLDRHKIVSTMRCKTRKESENREEQKRKDNQTSSGQKPTPWFLPQ 350
DmRdgC          VHGGFSDSTSLDLIKSIDRGKYVSILRP-------------------------------- 297
CePPEF          VHGGISDQTEVSKLDKIPRHRFQSVLRPPVNKGMESE----------------------- 404
TcPPEF          VHGGLVNIEDISRIQRFRQIP--------------------------------------- 474
TbPPEF          VHGGVPRRKGVRIEDISRIQRFRQIP---------------------------------- 473
LmPPEF          VHGGPPRRRGVSINDISRIQRFRQIP---------------------------------- 484
                :***                 * :

HsPPEF2         SRSLPSSPLRLGS-YKAQKTSRSSSIPCSGSLDGRELSR--QVRSSVELELERCRQQAGL 407
MmPPEF          SRSLPSSPFHLGSGFKAYKAGRSCSIPC-GSPNSKELSRRGQVRRSVDLELEQCRQQAGF 409
DmRdgC          -----PLTD--------------------------------------------------- 301
CePPEF          ------------------------------------------------------------
TcPPEF          ------------------------------------------------------------
TbPPEF          ------------------------------------------------------------
LmPPEF          ------------------------------------------------------------

HsPPEF2         LVTGEKEEPSRSASEADSEA--GELRKPTQEEWRQVVDILWSDPMAQEGCKANTIRGGGC 465
MmPPEF          LGIREKGESLPLAPDADCVADGGGVLEPTPEEWKQVVDILWSDPAAQEGCKANAVRGGGC 469
DmRdgC          ----------------------GEPLDKT--EWQQIFDIMWSDPQATMGCVPNTLRGAGV 337
CePPEF          ----------------------KENSAVNVDEWKQMLDIMWSDPKQNKGCWPNVFRGGGS 442
TcPPEF          -----------------------MPDYSQPEEDEIFQDLLWSDPVEDLQGWRESPRGAGV 511
TbPPEF          -----------------------MPDYSQPEEDEIFQDLLWSDPVEDLQGWRESHRGAGV 510
LmPPEF          -----------------------IPNYSQPEEDEIFQDMLWSDPVEDIKSWRESQRGAGV 521
                                               *  .  . *::****        :  **.*

HsPPEF2         YFGPDVTQQLLQKYNLQFLIRSHECKPEGYEFCHNRKVLTIFSASNYYEVGSNRGAYVKL 525
MmPPEF          YFGPDVTERLMEKYKLQLLIRSHECKPEGYEFCHNRKVLTIFSASNYYEVGSNRGAYVKL 529
DmRdgC          WFGPDVTDNFLQRHRLSYVIRSHECKPNGHEFMHDNKIITIFSASNYYAIGSNKGAYIRL 397
CePPEF          YFGADITASFLEKHGFRLLVRSHECKFEGYEFSHNNTCLTVFSASNYYETGSNRGAYVKF 502
TcPPEF          VFGADVTQEFLQNNGLELVIRSHEECLRGYEEHHDGKLLTVFSASNYDGPETNFGSFAVF 571
TbPPEF          EFGPDLTHEFLQSNGLELIIRSHCDCERGYEEFHDGKLVTVFSASNYNGPDTNYGSVAVF 570
LmPPEF          EFGSDVTMEFLENNKLELIVRSHEDCLSGYEEHHNHKLLTVFSASNYDGPNSNYGAICTF 581
                 **.*:*  :::   :  ::***     *:*  *:  .  :*:******    :* *:   :

HsPPEF2         G-PALTPHIVQYQ-ANKVTHT------------------------LTMRQRISRVEES-- 557
MmPPEF          G-PALTPHIVQYQ-ANKATHR------------------------LTMRQRISRVEES-- 561
DmRdgC          N-NQLMPHFVQYISAASQTKR------------------------LSFKQRMGIVESS-- 430
CePPEF          IGKSKQPHFVQYM--ASKTHRK-----------------------STLRERLGVVEES-- 535
TcPPEF          VGDNPEPSFHTYQVAEDDVEVQTLVDLGETFTPTLGRVS--SFATL-SQSKLLLR----- 623
TbPPEF          IGDNPEPSFSVHKVLEDDLEAHHHVDLGETQTGGLG-------MTLTSLSMLHLV----- 618
LmPPEF          IGDNPEPSYHTYQMFEDEYDDSQVVSLTDSFTLTAPNIARISSFTATVGSVGFGVSKSNF 641
                      *    :    .  .                                .
                                                 *  * *G* *   *
HsPPEF2         -------------ALRALREKLFAHSSDLLSEFKKHDADKVGLITLSDWAAAVESVLHLG 604
MmPPEF          -------------ALRALRQKLFAHSSDLLVEFRKRDPDESGVITLSDWATAVESVLHLG 608
DmRdgC          -------------ALKELAVRMRDHRDELEDEFRKYDPKDSGYISISHWCKVMENVTKLG 477
CePPEF          -------------AVKELKEKLSSFHTDLQKEFEIMDIEKSGKLPILKWSDCVERITGLN 582
TcPPEF          --RRAR-----DDVLRVLRERIYQRRHRLLAYFAKLDRTRKGSVWKIEWVEAMRNVLNLD 676
TbPPEF          --RPAIRRRTKDEVLRVLRERVYQRRHRLMAYFNKLDRTRKGSVWKIEWVEAMRNVLNTD 676
LmPPEF          MSRVLLHRRTKDNILRELRERIYQRRHRLLAYFSKLDRTNKGSLWMIEWVESMRNVLNLD 701
                              :: *  ::      *   *   *    * :   .*   :. :   .
```

```
HsPPEF2         LPWRMLRPQLVNSSADNM----------------LEYKSWLKNLAK-EQLSRENIQSSLL 647
MmPPEF          LPWRMLRPQLVNSSADNV----------------LEYRSWLDSLAK-EQLSRENIQSSLL 651
DmRdgC          LPWRLLRDKLAPGTDSQK----------------VNYNRTLDLLDTDVILEAEADGMSVM 521
CePPEF          LPWIALAPKVATLSEDGKY---------------VMYKEDRRIAQVGGTHAQEKD---IV 624
TcPPEF          LPWFFLRGYLVADDENTRVWYSHFLVKFHNFFQPLWLNDWIQSACYHLTQQQRANHRSQY 736
TbPPEF          LPWFFLRGYLAAEDDDGRIWYSLFLVKFHNKLQSLWLNEWISSARDRLIQQQRANHRSQY 736
LmPPEF          LPWYFLRGYLVEVDDHSRIWYAPFLNRFHNVLQDLWGEEWRQSVCARVLQQQRMNHRSQL 761
                ***  *   :.                       :  .              .

                                                      * * *G* *  *
HsPPEF2         ETLYRNR---------------------SNLETIFRIIDSDHSGFISLDEFRQTWKLFSS 686
MmPPEF          EKLYRNR---------------------SNLETIFRIIDSDHSGFISLDEFRQTWKLFSS 690
DmRdgC          DALYANK---------------------ASLVAIFNIIDADNSGEITLDEFETAIDLLVA 560
CePPEF          ESLYRHK---------------------STLETLFRFMDKDNSGQVSMKEFIDACEVLGK 663
TcPPEF          VAKAFNKEQVSYNEFCSVIRAIDYTMSEAQLFQLFVYLDEGGTGHIDGPKFVNMLSEMAA 796
TbPPEF          VANSFNREQVGYNEFCSVMRAIDYTMSDGHLFQLFVHFDERGVGFIDGRKFVHMLSEANT 796
LmPPEF          VSAAFNKEVVNYNEFCSVMRAIDYTTSDCQLFQLFESFDEKGNGHISGPEFLKKVQQIAN 821
                     ::                       *  :*  :*    * :   :*    .

                                              * * *G* *  *
HsPPEF2         ------------HMNIDITDDCICDLARSIDFNKDGHIDINEFLEAFRLVEKSCPE---- 730
MmPPEF          ------------HMSIDITDDGICDLARSIDFNKDGHIDINEFLEAFRLVEQSCLE---- 734
DmRdgC          ------------HMPGAYSKAEMLEKCRMMDLNGDGKVDLNEFLEAFRLSDLHRKEQQDE 608
CePPEF          ------------YTKRPLQTDYISQIAESIDFNKDGFIDLNELLEAFRLVDRPLLR---- 707
TcPPEF          -YPLSDTLRWDLGAMEQLQSVVIQGRSQLPYLFKVTSKDRALPKEKFMLGLAQLGRGMRK 855
TbPPEF          SHPGADPLRWDLEAMEQLQSVVIQGRGQLQYLFKVSTKDCVLTKESFMWGLEQLGRGMRR 856
LmPPEF          --GKPDPLRWDIDAMEQLQNIVIQGRSQLPSLFRVTSRDKALSRERFMAGMAQLGRCMRK 879
                                      :    .   :      *     * *        .

HsPPEF2         -------GDASECPQATN----------------------AKDSGCSSPGAH------- 753
MmPPEF          -------GHASACLQSTD----------------------TAESGHSSPGPC------- 757
DmRdgC          NIRRRSTGRPSVAKTATDPVTLLADKISKNTLVVEHDIDPTDCESKVIDPKKS------- 661
CePPEF          ------------------------------------------------------------
TcPPEF          QLTQQQKEKIYEFLAERAPPSG-VMFEHFLFMTAVFD---VQAMPRS-PAVVLADVNLFG 910
TbPPEF          QLTQQQKERIYEYLLERAPNGS-VLFGQFLFMTTVFD---NCTMSTS-SAIDLADINLVA 911
LmPPEF          QLTQPQREAIFDFMKQRVPEGEEVTFDMFLLCVYVFDRRTVAGMKSSYSLSDLNELGLAP 939

HsPPEF2         ---------------
MmPPEF          ---------------
DmRdgC          ---------------
CePPEF          ---------------
TcPPEF          NYLKNSSFSSMCK-- 923
TbPPEF          YCLRNCSFSSFVRD- 925
LmPPEF          ISFRNTSFSGLGTSR 954
```

Figure S2. Alignment of mammalian protein phosphatase-1 (PP1) and LmPPEF catalytic domains derived from homology modeling.

The model was generated using the automated comparative protein modelling server Swiss-Model and the structure visualised using DeepView (Swiss Pdb viewer; Guex N, Peitsch MC. SWISS-MODEL and the Swiss-PdbViewer: an environment for comparative protein modeling. Electrophoresis 1997;18:2714-2723). **α**-helices and β-sheets identified in the crystal structure of PP1 (Goldberg J, Huang HB, Kwon YG et al. Three-dimensional structure of the catalytic subunit of protein serine/threonine phosphatase-1. Nature 1995;376:745-753) are mapped onto the LmPPEF sequence. Residues highlighted in red are conserved residues that contribute to coordination of metal ions. Residues in blue contribute to the hydrogen bonding of the phosphorus oxygens. Residues in grey are not included in the model structure due to insufficient sequence identity.

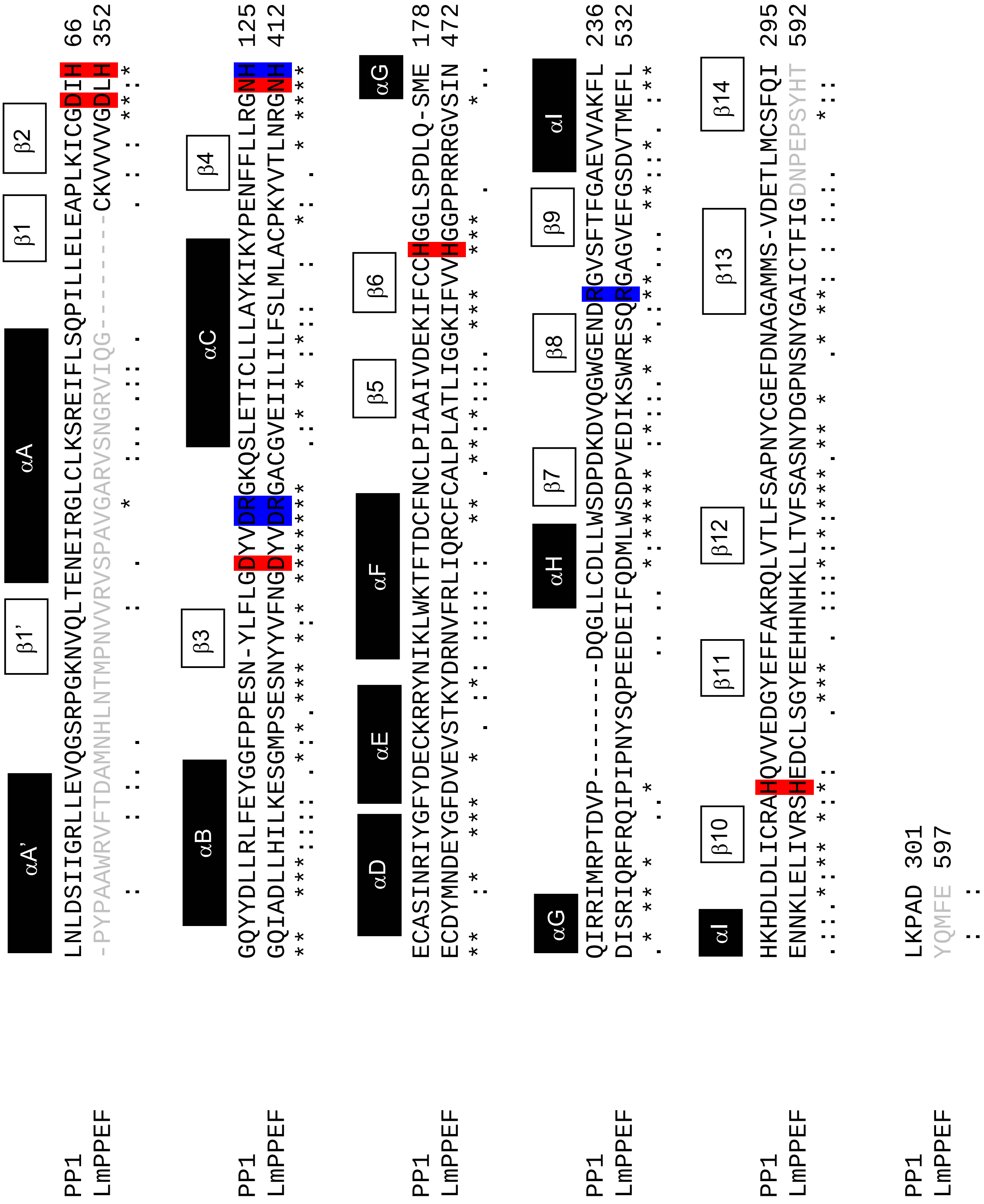

Table S1. *Leishmania N*-myristoylated proteins - high confidence predictions

N-myristoylated *L. major* proteins	N terminus	Description	Score	*T. brucei* orthologues	*T. cruzi* orthologues
LmjF28.1770	MGTHVSKVKS VNMDAWAEST	Arf GTPase activating protein, putative	0.99038119	Tb11.01.0920	Tc00.1047053503677.4 Tc00.1047053506223.10
LmjF04.0480	MGNVFSSWFE GLFSNKNASI	ADP-ribosylation factor, LmARF2 [1]	0.99034681	Tb09.160.5600	Tc00.1047053434945.4, Tc00.1047053509297.30, Tc00.1047053508197.20, Tc00.1047053508199.10
LmjF20.1230	MGSRISKEAA PVTFKNGKPT	hypothetical protein, conserved	0.99034442	Tb927.1.2160	Tc00.1047053508999.260, Tc00.1047053506563.170
LmjF04.0890	MGNAASSPRR APSASSSSSP	hypothetical protein, conserved	0.99029122	Tb09.211.0290	Tc00.1047053504255.30, Tc00.1047053510577.10, Tc00.1047053510575.239
LmjF27.1840	MGNRVSTAAA PFSLEDLEAL	hypothetical protein, conserved	0.99019717	Tb927.2.4280	Tc00.1047053509099.20, Tc00.1047053509321.10
LmjF16.1270	MGCRASASRN RESSHGSRAA	hypothetical protein, conserved	0.98972406	Tb927.8.5160	Tc00.1047053506575.50h Tc00.1047053511217.50 h
LmjF23.0170	MGGKASKAET RLTSEAADAL	hypothetical protein, conserved	0.98967251	-	Tc00.1047053505647.10
LmjF20.1270	MGCGSSAPKA DTSATNEEVR	hypothetical protein, conserved	0.98946806	-	Tc00.1047053509001.30, Tc00.1047053506563.120
LmjF03.0740	MGNSASSVGG AVPPQQQDQR	cytochrome c oxidase copper chaperone, putative	0.98937282	Tb927.3.2650	Tc00.1047053506691.64 Tc00.1047053508153.994
LmjF31.0440	MGCCNSKETK KPKEEVLQRE	cytoskeleton-associated protein CAP5.5, calpain-like	0.98936046	Tb04.1D20.740	Tc00.1047053509237.130, Tc00.1047053508555.60, Tc00.1047053511333.4
LmjF26.1440	MGSACSHGED ARHHRENRKN	hypothetical protein, conserved	0.98915106	Tb927.7.510	Tc00.1047053503723.10 Tc00.1047053506287.20
LmjF25.2060	MGARHSVPLR PNGQPSAQAL	hypothetical protein, conserved	0.989058	Tb927.3.2150	Tc00.1047053507081.50
LmjF32.1810	MGGGLSKSQL NVHYQGATEA	protein kinase, putative	0.98904117	-	-
LmjF31.2790	MGGWLSSLLG KKEVRILMVG	ADP-ribosylation factor, LmARF1 [1]	0.98864783	Tb09.211.4460 Tb09.211.4470 Tb09.211.4480 Tb09.211.4490	Tc00.1047053508415.40 Tc00.1047053508415.50 Tc00.1047053510755.50 Tc00.1047053510755.60
LmjF33.1035	MGCSASKSKE VKPSGGPGAS	hypothetical protein, conserved	0.98757771	Tb10.26.0245	Tc00.1047053509647.130
LmjF09.1550	MGSSSSKAKA TPAVSVPRYE	hypothetical protein, conserved	0.98742829	Tb11.01.5280	Tc00.1047053506753.40 Tc00.1047053510355.200
LmjF18.1440	MGGRISREQM NDYREVAKER	hypothetical protein, conserved	0.98726004	Tb10.389.1030	Tc00.1047053503455.20, Tc00.1047053511261.90
LmjF04.0790	MGSNVSVLLH ADTPVPNSVI	hypothetical protein, conserved	0.98650482	Tb09.211.0160	Tc00.1047053510579.50 Tc00.1047053510241.90
LmjF16.0930	MGFRESKPKL SQTAEKPEKS	hypothetical protein, conserved	0.98640037	Tb927.8.5430	Tc00.1047053509393.10 Tc00.1047053507491.140
LmjF24.1730	MGCSASTHAS PEADKDAPLK	protein kinase, putative	0.98564192	Tb08.30K1.240	Tc00.1047053506801.120, Tc00.1047053511907.240
LmjF36.0530	MGDMLAKPET QKFSTVFETS	protein phosphatase 2C-like protein	0.98457176	Tb10.70.2270	Tc00.1047053508659.40 Tc00.1047053510291.30
LmjF05.0840	MGNDLSAMSG GGRNLEVWSE	hypothetical protein, unknown function	0.98322847	-	-
LmjF06.0680	MGANGSKTAS SSSAVPAATT	hypothetical protein, conserved	0.98245443	Tb07.27E10.540	Tc00.1047053508175.39, Tc00.1047053506825.140
LmjF17.0370	MGQAASYAES SVQRALLRLK	hypothetical protein, unknown function	0.98211815	-	-
LmjF18.1150	MGGKNSTQRQ GAQLRNGNGG	hypothetical protein, conserved	0.98151093	Tb10.389.0570	Tc00.1047053503939.120, Tc00.1047053509805.80
LmjF23.0460	MGCKSSAEAK PSETRSPSAR	trypanothione synthetase, putative	0.98097255	Tb927.8.2410	Tc00.1047053504427.10 Tc00.1047053509331.134
LmjF30.0660	MGCLNSKSTA EKTGTSSPKR	hypothetical protein, conserved	0.9791003	Tb927.6.2090	Tc00.1047053507019.80
LmjF20.1260	MGSGFSRASA GDTSAPLPPC	hypothetical protein, unknown function	0.97836	-	-
LmjF10.1140	MGNRTSHEQP TATGVTDIEL	hypothetical protein, conserved	0.9765795	Tb927.8.4570	Tc00.1047053511409.40 Tc00.1047053509571.40
LmjF13.0680	MGRGYSASRS KSGRPTLRMK	hypothetical protein, conserved	0.9740294	Tb11.02.1670	Tc00.1047053506239.20, Tc00.1047053506177.10
LmjF32.2140	MGNSSSREWF DPYAVNTPLA	hypothetical protein, conserved	0.9735331	Tb11.01.7005	Tc00.1047053506425.50
LmjF20.1280	MGNKQSHAVG EFRYSGPTDD	calpain-like cysteine peptidase, putative	0.9732664	-	Tc00.1047053506563.110, Tc00.1047053509001.40
LmjF14.0300	MGSWDSAYED DAMDTRKLYC	hypothetical protein, conserved	0.9728694	Tb07.26A24.1030	Tc00.1047053504213.130, Tc00.1047053506985.20

LmjF21.0300	MGCSASPWAR WAAKVQHRCK	hypothetical protein,conserved	0.9719463	Tb10.70.5700	Tc00.1047053508949.20 Tc00.1047053510119.40
LmjF20.0700	MGATPSREAK PTIRSLRYGN	hypothetical protein, conserved	0.9705504	Tb927.1.1470	Tc00.1047053511425.49
LmjF31.1400	MGALPSHETH ERGLYSHRHG	hypothetical protein, conserved	0.9704316	Tb08.6H23.50	Tc00.1047053508965.100, Tc00.1047053509007.10
LmjF25.1750	MGCGYSTYSA QDDIVWKYGG	hypothetical protein, unknown function	0.9702202	-	Tc00.1047053477935.9 Tc00.1047053510091.119
LmjF23.1070	MGSSCTKDSA KEPQKSADKI	hydrophilic surface protein (HASPB)	0.9698957	-	-
LmjF36.0030	MGCTASKTKT PAANVASKGA	hypothetical protein, conserved	0.969785	Tb10.70.2830	Tc00.1047053506341.20, Tc00.1047053506507.20
LmjF36.4440	MGSSSSRQTP LPAGSADTAA	hypothetical protein, conserved	0.9683171	Tb10.6k15.0500	Tc00.1047053504147.90
LmjF31.1510	MGAVPSRETV EHWLSQPPFY	hypothetical protein, unknown function	0.9681234	Tb927.4.4580	-
LmjF03.0230	MGGAVSFCLQ KLNKVSEVEY	long chain fatty Acyl CoA synthetase, putative	0.9675061	Tb10.70.4200	Tc00.1047053503575.50, Tc00.1047053510943.33
LmjF20.1490	MGAANSCTCS ETKYRFKDFI	hypothetical protein, conserved	0.9669213	Tb927.1.3290	Tc00.1047053503663.4 Tc00.1047053510635.60
LmjF10.1230	MGSNASHTEP QVPRECTMVA	hypothetical protein	0.9662321	-	-
LmjF33.2380	MGQDQSRAKA CQLGQAETGG	Golgi reassembly stacking protein (GRASP homologue), putative	0.96612	Tb11.02.0260	Tc00.1047053504153.320, Tc00.1047053506301.20
LmjF30.0070	MGGHGSMNPN AQITTRDAAG	hypothetical protein, conserved	0.9600807	Tb927.6.1450	Tc00.1047053505807.54 Tc00.1047053509033.70
LmjF23.1490	MGGATSKDRY DRAAATGILT	hypothetical protein, unknown function	0.958393	-	-
LmjF31.2280	MGQWLASAFS SLMGKKEVRI	ADP-ribosylation factor, LmARF3 [1]	0.9567458	-	-
LmjF27.1230	MGAAAAKPVR EAPVRYVAKQ	hypothetical protein, conserved	0.95649	Tb11.39.0010	Tc00.1047053509837.20
LmjF31.0920	MGNRQSSVPL EGEGHPEVFY	sodium stibogluconate resistance protein, putative	0.9509094	Tb07.27E10.660	Tc00.1047053506681.40, Tc00.1047053506825.40
LmjF31.0960	MGNRQSSVPL EGEGHPEVFY	sodium stibogluconate resistance protein, putative	0.9509094	Tb07.27E10.660	Tc00.1047053506681.40, Tc00.1047053506825.40
LmjF31.0930	MGNRQSSVPL EGEGHPEVFY	sodium stibogluconate resistance protein, putative	0.9509094	Tb07.27E10.660	Tc00.1047053506681.40, Tc00.1047053506825.40
LmjF24.1360	MGSSSSTLET AKLVRTLLSE	hypothetical protein, conserved	0.9505801	Tb927.8.6860	Tc00.1047053507991.80, Tc00.1047053504035.120
LmjF04.0340	MGQLFTSLWS MFNGNKSYKL	ADP-ribosylation factor, LmARLX [1]	0.9493244	Tb09.160.5300	Tc00.1047053506627.60
LmjF21.0861	MGNSSSRSGS SLSHTAVGGS	hypothetical protein	0.9478317	-	-
LmjF20.1310	MGCGASSENS SVTYVNGRPT	calpain-like cysteine peptidase, SMP-1 [2]	0.9157358	Tb927.1.2260, Tb927.1.2230	Tc00.1047053509003.30, Tc00.1047053509003.40, Tc00.1047053506563.70, Tc00.1047053506563.79
LmjF36.5660	MGQRGSRVSP VVASASDPPS	hypothetical protein, conserved	0.914911	Tb10.6k15.1280	Tc00.1047053503525.40 Tc00.1047053507825.30
LmjF12.0660	MGCDSSKEGV ASNTASASSR	serine/threonine protein phosphatase-like protein	0.9116546	Tb927.1.4050	Tc00.1047053506529.380, Tc00.1047053510889.80
LmjF20.0705	MGAVPSRECT SLFSYFRGSY	hypothetical protein, conserved	0.8998901	Tb927.1.1500	Tc00.1047053511427.19, Tc00.1047053504209.10
LmjF19.1280	MGTCPSISQS EVGIVETCGR	hypothetical protein, conserved	0.8959736	-	Tc00.1047053507659.10, Tc00.1047053507585.20
LmjF03.0520	MGDRDSCTQR DSRADRYGGD	RNA binding protein, putative	0.8885153	Tb10.70.3950	Tc00.1047053510265.40 Tc00.1047053510311.50
LmjF36.4600	MGVGASALTL SLSFHLLRPF	hypothetical protein, conserved	0.8857702	Tb10.6k15.0340	Tc00.1047053503847.50 Tc00.1047053504103.60

All protein entries identified by GeneDB accession numbers. *L. major* specific proteins are highlighted or in red text; identical highlighting colour represents related function. Not all *T. brucei/T. cruzi* orthologues are predicted as N-myristoylated with high confidence (see Tables S2 and S3).
References: [1] Price HP, Panethymitaki C, Goulding D. Functional analysis of TbARL1, an N-myristoylated Golgi protein essential for viability in bloodstream trypanosomes. J Cell Sci 2005;118:831-841; [2] Tull D, Vince JE, Callaghan JM. SMP-1, a member of a new family of small myristoylated proteins in kinetoplastid parasites, is targeted to the flagellum membrane in Leishmania. Mol Biol Cell 2004;15:4775-4786.

Table S2. *T. brucei* *N*-myristoylated proteins - high confidence predictions

Accession no.	N-terminus	Description	Score
Tb10.70.2270	MGETLSKPVT EKHTSTFETS	protein phosphatase 2C-like, putative	0.99037937
Tb11.02.0010	MGCGGSKPNA VSRDVEEKAL	hypothetical protein, conserved	0.9901967
Tb09.160.5300	MGQLISGLWS VFNPNRHYKL	ADP-ribosylation factor, TbARLX [1]	0.98999036
Tb09.211.0160	MGSDLSIVLR TCVAAPDCTK	hypothetical protein, conserved	0.9898953
Tb10.70.5540	MGGSYSTVKT QRELRNADLK	ubiquitin hydrolase, putative	0.98970955
Tb11.39.0010	MGAARSKPAR EAPVRYVAKQ	hypothetical protein, conserved	0.98970702
Tb08.5H5.70	MGNNGSSTVR PPASQTKRQG	hypothetical protein, conserved	0.98962292
Tb08.26E13.20	MGCGGSKTST VEFINGQPTV	flagellar calcium-binding protein	0.98957076
Tb927.1.2230	MGCGGSKTST VEFINGQPTV	calpain-like protein fragment, putative	0.98933103
Tb09.160.5600	MGNVLSWFEG LFSKKDATIL	ADP-ribosylation factor, TbARF2 [1]	0.9893292
Tb09.160.1330	MGSSMSCLEV GSDSRCFGLP	hypothetical protein, conserved	0.98922819
Tb08.30K1.240	MGCGASSRVV ESDDRERHKS	Ser/ Thr protein kinase, putative	0.98919997
Tb08.5H5.30	MGCSASKDTT NSKDGAASKG	flagellar calcium-binding protein TB-44A	0.98895118
Tb11.53.0001	MGTTMSGNQG RKRQRISTPE	hypothetical protein, leucine-rich repeats	0.98895106
Tb11.46.0013	MGSECSRHSN QTELRDERFC	hypothetical protein, conserved	0.98884212
Tb927.1.1020	MGCAGSQQQK PRSNQNIRTK	hypothetical protein, conserved	0.98871979
Tb927.1.2150	MGLFLSKQGE GQLLAEPTMQ	calpain-like protein fragment, putative	0.9886871
Tb08.5H5.50	MGCSGSKNTT NSKDGAASKG	flagellar calcium-binding protein	0.98854366
Tb11.02.1220	MGQAGGKEQK RQQEKWEPPV	proteasome regulatory ATPase subunit 2	0.98821444
Tb04.3M17.490	MGSAAGKIGE KKHDRGEKLR	hypothetical protein	0.98766599
Tb10.26.0245	MGCGDSKVRQ GGPSRPPPPR	hypothetical protein, conserved	0.98653345
Tb08.26A17.60	MGCGGSAPAN KDRHVGADPP	hypothetical protein	0.98639942
Tb08.26A17.40	MGCGGSAPAN KDRHVGADPP	hypothetical protein	0.98639942
Tb10.70.5250	MGGCVSTALK VGAETVAEGH	metacaspase MCA4	0.98619341
Tb08.26A17.50	MGCGGSAPAN KNRHVGADPP	hypothetical protein	0.98570268
Tb08.26N11.570	MGCGGSAPAN KDKHVGADPP	hypothetical protein	0.98558802
Tb04.1D20.740	MGCGGSKVKP QPPQAAAPPP	cytoskeleton-associated protein CAP5.5 [2]	0.98461163

Tb927.1.2120	MGCGASSTQS EDGMHEPIPP	calpain, putative	0.98448321
Tb10.389.0570	MGGSSSANRN NRPPNRVSGS	hypothetical protein	0.98377993
Tb07.27E10.540	MGTTASTDSA DSPFSVRSVD	hypothetical protein	0.98374526
Tb04.3I12.600	MGALPSRETQ RRGLYSHRHG	hypothetical protein	0.98372966
Tb10.26.0825	MGTIFSNVRT HEEVPPEVKD	hypothetical protein, conserved	0.98300802
Tb04.26G5.164	MGQDQSMAFA NDTISGEQYR	hypothetical protein, conserved	0.98105446
Tb10.70.4650	MGNCLCCRDK HKHEPEPEVV	hypothetical protein, conserved	0.9793612
Tb07.15M23.530	MGCGDSRVRA SAQPQHQSHP	hypothetical protein	0.9772366
Tb08.6H23.50	MGALPSRETQ HRGLYSHRHG	hypothetical protein	0.9745772
Tb11.02.3780	MGGCTSRGLS EEKLACYSHR	phosphoinositide-specific phospholipase C, putative	0.9720402
Tb09.160.0950	MGQRGSSPGI CNYTNNTEKD	hypothetical protein, conserved	0.9690323
Tb10.61.1480	MGGGACKMER HDVQPRVSSS	hypothetical protein, conserved	0.9645248
Tb07.10C21.410	MGCNLSSSTK NVNPFGVKGV	hypothetical protein, conserved	0.9636023
Tb07.8P12.840	MGGGGSIPRK KATSARQVST	hypothetical protein, conserved	0.9635237
Tb11.01.1020	MGCQQSGVRM SPQDLSDRTP	hypothetical protein, conserved	0.9606532
Tb10.6k15.2160	MGCSASFSFF PRCDTATNRI	hypothetical protein	0.9596866
Tb08.4A8.170	MGQKSSTAAN EGRPRSYYLV	hypothetical protein, conserved	0.9596286
Tb11.02.1670	MGRGYSNSRS RSLRPTLRMK	hypothetical protein, conserved	0.9571304
Tb11.01.1420	MGNAPSFCCC CFPFFSQRRA	hypothetical protein, conserved	0.9524696
Tb07.8P12.730	MGSGCSTESS TDVRCSFVGL	hypothetical protein, conserved	0.9447624
Tb08.26A17.310	MGQHSAKEFR GSRRGESNSG	hypothetical protein, conserved	0.9445662
Tb03.3K10.420	MGCGIATLFP YSKGSSVSPT	hypothetical protein, conserved	0.9401887
Tb10.70.6250	MGSGGSPLLL SHCWWCFPCA	hypothetical protein	0.939623
Tb07.2F2.550	MGALVSQIKS LLGILPADRK	ADP-ribosylation factor, TbARL1 [1]	0.9383506
Tb10.6k15.0500	MGSSSSSAND SSSANMDAAS	hypothetical protein, conserved	0.9346623
Tb927.1.1420	MGAFPSHDAP ELIYSMLVLR	hypothetical protein, conserved	0.9297298
Tb10.6k15.1280	MGSFTSCDQR PTASTTAAPH	hypothetical protein, conserved	0.9243812
Tb10.6k15.3950	MGSTSSACRR LETACRTGEN	hypothetical protein, conserved	0.917454
Tb08.28F14.40	MGDSISPSEL LPGHAGEEHF	RNA-binding protein RBP10, putative	0.9113934

Tb06.4M18.340	MGLCNSKSFE RDQLAPPPGN	hypothetical protein, conserved	0.9047419
Tb10.389.1030	MGYTFSRAQM EDYKELVKGR	hypothetical protein, conserved	0.8943619
Tb04.3I12.520	MGAVPSRESL SRGLCRLPDY	hypothetical protein	0.8882656
Tb09.160.4560	MGFGSSKPKN KPSKADSKNS	arginine kinase	0.874727
Tb927.1.4050	MGCSTSKGDP LRTNKGAGDG	Ser/Thr protein phosphatase (PPEF)	0.8666006
Tb08.6H23.30	MGAAPSRETL RRGLYNVPTQ	hypothetical protein	0.8537426

T. brucei proteins also predicted as N-myristoylated with high confidence in *L. major* are in red text.

References: [1] Price HP, Panethymitaki C, Goulding D. Functional analysis of TbARL1, an N-myristoylated Golgi protein essential for viability in bloodstream trypanosomes. J Cell Sci 2005;118:831-841; [2] Hertz-Fowler C, Ersfeld K, Gull K. CAP5.5, a life-cycle-regulated, cytoskeleton-associated protein is a member of a novel family of calpain-related proteins in Trypanosoma brucei. Mol Biochem Parasitol. 2001;116:25-34.

Table S3. *T. cruzi N*-myristoylated proteins - high confidence predictions

Accession no.	N-terminus	Description	Score
Tc00.1047053507011.80	MGTNGSKDER WQRDSEAPSC	hypothetical protein	0.99024417
Tc00.1047053507005.40	MGNNGSKGER WQRDSEAPSC	hypothetical protein	0.99023844
Tc00.1047053509237.130	MGCCASKEKQ PRPGAAAEPT	cytoskeleton-associated protein CAP5.5, putative	0.99022871
Tc00.1047053504933.30	MGSVFSKKSE EPPKAQTGKA	hypothetical protein	0.9902219
Tc00.1047053506627.60	MGTLFSSFWS LFVPSRNYKL	ADP-ribosylation factor, TcARLX	0.99019659
Tc00.1047053510839.20	MGSILSKKSE EPPKAQTGKA	hypothetical protein	0.99017361
Tc00.1047053507803.30	MGGGISNANQ RENAQDDDGN	hypothetical protein	0.99007086
Tc00.1047053507063.290	MGGGISNTNQ RENAQDDNVN	sericin MG-2 -greater wax moth (fragments), putative	0.99000427
Tc00.1047053503409.10	MGCSQSKTRT KEWGGPQQRR	hypothetical protein, conserved	0.98992987
Tc00.1047053509965.20	MGCGVSRQPF STELRPLRAT	hypothetical protein	0.98967212
Tc00.1047053510901.190	MGCRHSGPRM PPLEQQQGHS	hypothetical protein, conserved	0.98954869
Tc00.1047053506681.40	MGNGASVGEN RHSQLFQQGY	Sodium stibogluconate resistance protein, putative	0.98940584
Tc00.1047053506825.40	MGNGASVGEN RHSQLFQQGY	Sodium stibogluconate resistance protein, putative	0.98940584
Tc00.1047053508175.60	MGCSNSKNQK GREDKVDPHE	hypothetical protein	0.98933805
Tc00.1047053506825.160	MGCSNSKNQK EREDKVDPHE	hypothetical protein	0.98932215
Tc00.1047053511333.4	MGCCASKEKQ PRPEAAAGPT	hypothetical protein	0.98916881
Tc00.1047053508555.60	MGCCASKEKQ PRPEAAAGPT	cytoskeleton-associated protein CAP5.5, putative	0.98916881
Tc00.1047053507891.47	MGACGSKDST SDKGLASDKD	hypothetical protein	0.98916826
Tc00.1047053507891.20	MGCCGSKEDT CGLVYMEMRR	hypothetical protein	0.98915021
Tc00.1047053506749.10	MGCCGSKEDT CGLVYMEMLR	hypothetical protein	0.98915021
Tc00.1047053511421.150	MGCKESSLRS PDVKKKDDEL	hypothetical protein, conserved	0.98894311
Tc00.1047053507623.50	MGAVHSQAQE KQGMEEKYNN	oxidoreductase, putative	0.98893028
Tc00.1047053510729.60	MGCVLSNQKL LQNSNRQCSS	hypothetical protein, conserved	0.98891372
Tc00.1047053506885.354	MGCVLSNQKL LQNSNRQCNS	hypothetical protein	0.98891372
Tc00.1047053508921.30	MGGGVSTRKQ MQDDRNVWNS	MCAK-like kinesin, putative	0.98887981
Tc00.1047053507023.90	MGGGLSRQEE LFVLGQWPEL	hypothetical protein, conserved	0.98861717

Tc00.1047053509785.40	MWRCCGRLHV ALLRARWASA	RHS1 protein, putative	0.98844714
Tc00.1047053511261.90	MGQGFSRAQM EEYKELVKDR	hypothetical protein, conserved	0.98817444
Tc00.1047053503455.20	MGQGFSRAQM EEYKELVKDR	hypothetical protein, conserved	0.98817444
Tc00.1047053506287.100	MGSGGSTSLG GRITTVKEKK	hypothetical protein, conserved	0.98805283
Tc00.1047053508881.150	MGSGGSTSLG GRITTVKEKK	hypothetical protein, conserved	0.98805283
Tc00.1047053511907.240	MGCGASSRVK VPPVCRRSSS	Ser/Thr protein kinase, putative	0.98791753
Tc00.1047053508999.230	MGNGVSSTKG KSGNNNIVTT	calpain-like protease, putative	0.98774301
Tc00.1047053503939.120	MGANSSRERR SQRRSQSNAV	hypothetical protein, conserved	0.98752071
Tc00.1047053511123.20	GRASATPNAA GTVRPNSRDC	RHS1 protein	0.98740598
Tc00.1047053508277.220	MGSAFSGEMS DTRELASVFL	hypothetical protein	0.98719425
Tc00.1047053509945.30	MGSAFSGEMS DTRELASVFL	hypothetical protein	0.98719425
Tc00.1047053509647.130	MGCKNSKARE PPADTVTAAQ	hypothetical protein	0.98686375
Tc00.1047053508869.50	MMMCRLLCAL LVLALCCCPS	hypothetical protein	0.98673219
Tc00.1047053511071.90	MGARHSCPVP SAKAPVPSPV	hypothetical protein	0.98657112
Tc00.1047053508999.250	MGSSTSKHNP RELLATPAMR	hypothetical protein, conserved	0.98651511
Tc00.1047053506749.20	MGACGSKGST SDKGLASDKD	flagellar calcium-binding protein [2]	0.98597729
Tc00.1047053508141.70	MGGGASTRKQ MQDDRNVWNS	MCAK-like kinesin, putative	0.98595934
Tc00.1047053506925.220	MGTSMCVPRR RRDDSPLSYF	hypothetical protein, conserved	0.98575612
Tc00.1047053506563.180	MGSSTSKHNP KELLATPAMR	hypothetical protein, conserved	0.98542494
Tc00.1047053508813.80	MGGRTSELQS QTPPSSLEQN	hypothetical protein, conserved	0.98448939
Tc00.1047053508917.20	MGCGASRNVK ADADVSLPVF	hypothetical protein, conserved	0.98407297
Tc00.1047053508355.370	MGETCSKTAN AAALENRRIQ	hypothetical protein, conserved	0.98358552
Tc00.1047053508357.30	MGETCSKTAN AAALENRRIQ	hypothetical protein, conserved	0.98358552
Tc00.1047053508999.220	MGCGASTTQR PTTVAPEWNA	calpain, putative	0.98329955
Tc00.1047053506513.150	MGCGASRKVK ADADVSLPVF	hypothetical protein, conserved	0.98302486
Tc00.1047053509007.10	MGALPSRETQ RRGVYSHRHG	hypothetical protein	0.98264043
Tc00.1047053508965.100	MGALPSRETQ RRGVYSHRHG	hypothetical protein	0.98264043
Tc00.1047053510575.239	MGAAASSGRR VPRATSASPY	hypothetical protein	0.98248632

Tc00.1047053505943.20	MGAVNSQAQE GQGMEEKDNN	oxidoreductase, putative	0.98228996
Tc00.1047053506563.110	MGCGSSKSKT KADDFVNGRP	calpain-like cysteine peptidase, putative	0.98185002
Tc00.1047053509837.20	MGAARSRPAR EAPVRYVAKQ	hypothetical protein, conserved	0.98157868
Tc00.1047053506363.130	MGDEGSGSKE ESSGVPDDRV	hypothetical protein, conserved	0.98092273
Tc00.1047053504153.320	MGQEGSKVKE TLKGICGLQV	Golgi reassembly stacking protein (GRASP homologue), putative	0.98086905
Tc00.1047053506301.20	MGQEGSKVKE TLKGICGLQV	Golgi reassembly stacking protein (GRASP homologue), putative	0.98086905
Tc00.1047053503955.50	MGSVPSREFH ESPDALFFTR	hypothetical protein	0.98036138
Tc00.1047053507063.60	MGSVPSREFH ESPDALFFTR	hypothetical protein	0.98036138
Tc00.1047053507485.80	MGQSNSWALA SDTIKGEQYR	hypothetical protein, conserved	0.9800526
Tc00.1047053507159.14	MGQSNSWALA SDTIKGEQYR	hypothetical protein	0.9800526
Tc00.1047053511389.50	MGAVPSRETQ RRGLYFPRAP	hypothetical protein	0.9795058
Tc00.1047053506825.150	MGNCCSSNSK KRRQPEEAHV	hypothetical protein	0.9782967
Tc00.1047053506563.200	MGCGASTTQK PTTVAPEWNV	calpain, putative	0.9779467
Tc00.1047053509001.40	MGCGSSKSKT KADGFVNGRP	calpain-like cysteine peptidase, putative	0.9766528
Tc00.1047053509805.80	MGVNSSRERR SQRRSQSNAV	hypothetical protein, conserved	0.9765709
Tc00.1047053511903.10	MGAHHSFPVP SAKAPVPSPV	hypothetical protein	0.9760431
Tc00.1047053508175.50	MGNCCSSNSK KRRLPKEAHV	hypothetical protein	0.9751612
Tc00.1047053504147.90	MGGGSSKPEL KVGEKGAIAS	hypothetical protein, conserved	0.9748331
Tc00.1047053506563.190	MGNGGSSTKG KSGNNNIVTT	calpain-like cysteine peptidase, putative	0.9736424
Tc00.1047053509393.10	MGIRPSKTQG QRKSSVETPK	hypothetical protein, conserved	0.9721153
Tc00.1047053508999.260	MGSSSSKLTY ENGKPTFGEA	hypothetical protein, conserved	0.9716336
Tc00.1047053506563.170	MGSSSSKLTY ENGKPSFGEI	hypothetical protein, conserved	0.9708798
Tc00.1047053507669.239	IDKLDKAASE ALSAMGPSMT	hypothetical protein	0.9696272
Tc00.1047053506563.79	MGCGASSKPS TVEYKNGKPS	hypothetical protein,	0.96946
Tc00.1047053509003.30	MGCGASSKPS TVEYKNGKPS	calpain-like cysteine peptidase, putative	0.96946
Tc00.1047053509641.10	MGQLLSFNAT HEEVPEDVKE	hypothetical protein	0.9693288
Tc00.1047053511217.50	MGCRQSVAKR PVDPQEAAAA	hypothetical protein, conserved	0.9691444

Tc00.1047053510151.19	MGGLISTLMD IRNSMSLVPH	hypothetical protein,	0.9685729
Tc00.1047053504069.53	MFSSKTEYDR GVNTFSPEGR	hypothetical protein	0.9684737
Tc00.1047053506829.110	MGGLISTLMD LRNSMSLVPH	fatty acyl CoA synthetase 2	0.9677306
Tc00.1047053506575.50	MGCRQSVAKR PVAPQEAAAA	hypothetical protein, conserved	0.9676109
Tc00.1047053509233.239	GSCVPLQRYV DETGDVQTPV	hypothetical protein	0.9660238
Tc00.1047053510579.50	MGSDLSVVVQ ACAPVPHVIH	hypothetical protein, conserved	0.9625061
Tc00.1047053510241.90	MGSDLSVVVQ ACAPVPHVIH	hypothetical protein, conserved	0.9625061
Tc00.1047053511425.49	MGGVPSRDAC PTFFSLRYGS	hypothetical protein	0.9568907
Tc00.1047053506513.60	MGAWISQLKA ALGFLPADKK	ADP-ribosylation factor, TcARL1A [1]	0.9552285
Tc00.1047053507491.140	MGIRPSKTQG QRKSSVEAPK	hypothetical protein, conserved	0.9548726
Tc00.1047053506563.50	MGGGASSTSC DDPTTTSRIK	hypothetical protein, conserved	0.9507879
Tc00.1047053509003.60	MGGGASSTSC DDPTTTSRIK	hypothetical protein, conserved	0.9507879
Tc00.1047053506529.380	MGCTNTKESS ERGGGDASEL	serine/threonine protein phosphatase, putative	0.948353
Tc00.1047053506801.120	MGCGASSRVK VPPGCRGLSS	Ser/Thr protein kinase, putative	0.9430079
Tc00.1047053510889.80	MGCTNTKESS ERGGGNASEL	serine/threonine protein phosphatase, putative	0.9423383
Tc00.1047053508199.10	MGAVMSWFDG LFAKKDATIL	ADP-ribosylation factor, TcARF2 [1]	0.9356146
Tc00.1047053509297.30	MGAVMSWFDG LFAKKDATIL	ADP-ribosylation factor, TcARF2 [1]	0.9356146
Tc00.1047053508197.20	MGAVMSWFDG LFAKKDATIL	ADP-ribosylation factor, TcARF2 [1]	0.9356146
Tc00.1047053434945.4	MGAVMSWFDG LFAKKDATIL	hypothetical protein	0.9356146
Tc00.1047053510755.60	MGQWLASAFK SLLGKQEVRI	ADP-ribosylation factor, TcARF1 [1]	0.9285932
Tc00.1047053510755.50	MGQWLASAFK SLLGKQEVRI	ADP-ribosylation factor, TcARF1 [1]	0.9285932
Tc00.1047053508415.50	MGQWLASAFK SLLGKQEVRI	ADP-ribosylation factor, TcARF1 [1]	0.9285932
Tc00.1047053508415.40	MGQWLASAFK SLLGKQEVRI	ADP-ribosylation factor, TcARF1 [1]	0.9285932
Tc00.1047053510329.360	MGFHHSKSLS SSTCFDAVIR	hypothetical protein, conserved	0.9280773
Tc00.1047053507257.150	MGFHHSKSLS SSTCFDAVIR	hypothetical protein, conserved	0.9280773
Tc00.1047053511319.30	MGEIFSSVWA SEGNIARQCV	hypothetical protein	0.9264863
Tc00.1047053506239.20	MGRGYSNSRS KSARPTLRMK	hypothetical protein, conserved	0.9119683
Tc00.1047053506177.10	MGRGYSNSRS KSARPTLRMK	hypothetical protein, conserved	0.9119683
